# Recent Applications of Hydantoins in Drug Discovery: Updates (2019~Present)

**DOI:** 10.3390/molecules31050779

**Published:** 2026-02-26

**Authors:** Jyoti Dnyaneshwar Palkhede, Eo-Jin Park, Om Darlami, Dongyun Shin

**Affiliations:** Gachon Institute of Pharmaceutical Sciences and College of Pharmacy, Gachon University, Hambakmoe-ro 191, Yeonsu-gu, Incheon 21936, Republic of Korea

**Keywords:** hydantoin, medicinal chemistry, privileged scaffold, drug discovery, pharmacokinetic profiles

## Abstract

Hydantoins, exemplified by the imidazolidine-2,4-dione core, are privileged scaffolds in medicinal chemistry due to their compact structure, versatile hydrogen-bonding capacity, ability to fine-tune physicochemical properties for drug-like molecules, and potential to engage a diverse array of biological targets. This review highlights major advances in hydantoin-based drug discovery since 2019, emphasizing their evolving applications in oncology; neurology; infectious diseases; and cardiovascular, metabolic, and immune disorders. Recent studies demonstrate their success as kinase inhibitors, androgen receptor antagonists, and metalloprotease inhibitors, and emerging roles in modulating sterol isomerase, glycogen synthase kinase-3β, and ADAMTS family enzymes. Novel hybrid scaffolds—such as catechol–hydantoins, β-carboline–hydantoins, and spirocyclic thiohydantoins—have yielded potent and selective anticancer and antiviral leads. The discovery of BAY-9835 and GLPG1972 underscores the clinical potential of hydantoin-based metalloproteinase inhibitors in cardiovascular and osteoarthritic conditions. Furthermore, new antimicrobial, antimalarial, and antileishmanial derivatives illustrate the scaffold’s capacity to address multidrug resistance and neglected tropical diseases. Advances in computational design, stereochemical optimization, and hybridization strategies have expanded the structural and functional diversity of hydantoins, enhancing their target selectivity and pharmacokinetic profiles. Overall, hydantoins and their analogs remain at the forefront of small-molecule drug discovery, offering rich prospects for therapeutic innovation in diverse disease areas.

## 1. Introduction

Hydantoins, or imidazolidine-2,4-diones, have long occupied a prominent position in medicinal chemistry due to their compact, conformationally constrained, and pharmacologically versatile five-membered ring structure. The hydantoin nucleus, composed of a ureide moiety fused into a cyclic imide system, exhibits a unique combination of hydrogen bond donors and acceptors, lipophilic and polar regions, and stereogenic centers—features that facilitate diverse interactions with a wide array of biological targets. Their incorporation into drug-like molecules enables fine-tuning of physicochemical and pharmacokinetic properties, rendering hydantoins a privileged scaffold in small-molecule drug development [1,2,3].

In addition to its favorable pharmacophoric properties, the hydantoin scaffold is highly versatile from a synthetic and medicinal chemistry perspective. A wide range of post-cyclization modifications can be readily introduced on an already formed hydantoin ring, including N-alkylation or N-acylation at the imide nitrogens and functionalization at the C-5 position, for example, through Knoevenagel-type condensations leading to benzylidene derivatives.

**Figure 1:**
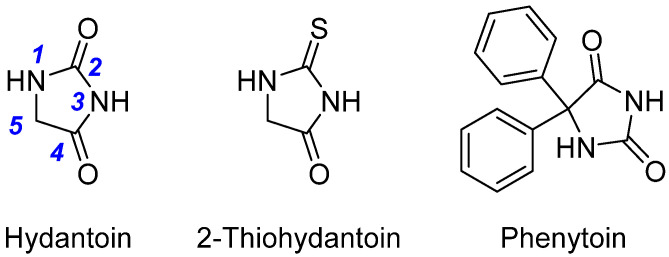
Structure of hydantoin, 2-thiohydantoin and phenytoin.

The therapeutic significance of hydantoins was first recognized with the introduction of phenytoin (diphenylhydantoin) as an antiepileptic agent in the 1930s [4] (Figure 1). Since then, both natural and synthetic hydantoin derivatives have demonstrated a broad spectrum of biological activities, including anticonvulsant [5,6], anti-inflammatory [7,8], antimicrobial [9,10], anticancer [11,12,13,14], antidiabetic [15,16], and antiviral [17,18,19]. Notably, the structural plasticity of the hydantoin core—particularly its capacity for N- and C-substitutions—has enabled medicinal chemists to design a wide variety of bioactive compounds with distinct modes of action.

In our previous review [1], we provided a comprehensive survey of the medicinal chemistry landscape of hydantoin and thiohydantoin derivatives up to that time. This earlier work documented their synthetic approaches, biological activities, and SARs, with a focus on their potential for lead optimization and target-specific drug design. Since then, the field has continued to evolve rapidly, catalyzed by emerging therapeutic targets, unmet medical needs, and innovations in synthetic and computational methodologies.

In recent years, the continued evolution of medicinal chemistry tools and drug discovery paradigms has further expanded the landscape of hydantoin-based compounds. This includes the integration of hydantoin cores into hybrid scaffolds, the design of multi-target ligands, and the incorporation of hydantoins into peptidomimetic and macrocyclic frameworks [20]. Additionally, the increasing application of computational modeling and structure-based drug design has enabled more rational exploration of hydantoin derivatives as inhibitors, modulators, or ligands for emerging and challenging targets [21].

In this updated review, we aim to provide an updated and critical overview of the most recent developments in the application of hydantoins in drug discovery, covering the period from 2019 to the present. Particular emphasis is placed on new pharmacological activities and target classes, and we hope to offer insights into the evolving role of hydantoins in modern medicinal chemistry and their prospects for future therapeutic innovation.

## 2. Pharmacological Applications of Hydantoins and Thiohydantoins

### 2.1. Anticancer Activity

**Figure 2:**
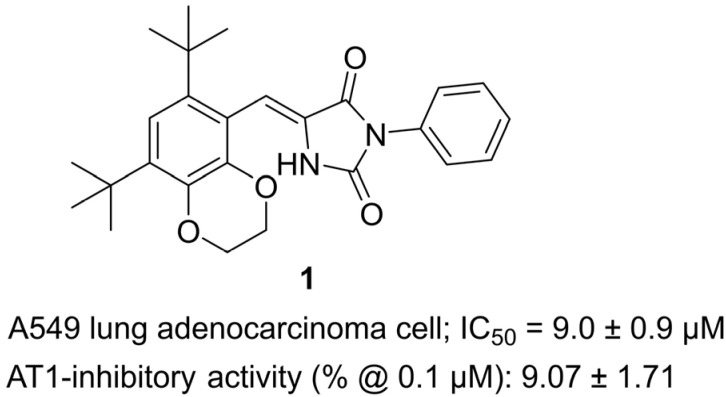
5-Benzylidene hydantoin **1**.

In 2025, Bai and colleagues synthesized a novel series of catechol–hydantoin hybrids as part of a hybrid pharmacophore approach that combines the antioxidant, metal-chelating, and signaling-modulating properties of catechols with the pharmacological versatility of the imidazolidine-2,4-dione (hydantoin) scaffold [22] (Figure 2). The design rationale was to exploit catechol derivatives (notably 3,5-di-tert-butylcatechol, 3,5-DTBC), whose steric bulk increases kinetic stability and bioavailability, and to fuse them with hydantoin cores to yield multifunctional bioactive molecules. Among the prepared library, 5-benzylidene hydantoin **1** was identified as a representative analogue. Structurally, it integrates a bulky tert-butyl-substituted catechol moiety linked through a benzylidene bridge to the hydantoin nucleus, enhancing steric protection and lipophilicity while retaining the hydrogen-bonding capacity of the hydantoin ring. In biological assays, hydantoin **1** demonstrated cytotoxicity against A549 lung adenocarcinoma cells with an IC_50_ of 9 ± 0.9 µM, indicating moderate antiproliferative potential. It also showed weak AT1 receptor inhibitory activity, reducing the angiotensin II–mediated contractile response by ~9% at 0.1 µM, about five-fold weaker than losartan, suggesting only limited direct renin–angiotensin system modulation. Taken together, these findings show that catechol–hydantoin hybrids can achieve dual anticancer and cardiovascular-relevant bioactivities, though in this early example cytotoxicity is more prominent than AT1 antagonism. hydantoin **1** thus provides a proof of concept for hybrid scaffold design, encouraging further optimization to balance anticancer potency with selective AT1 receptor inhibition.

**Figure 3:**
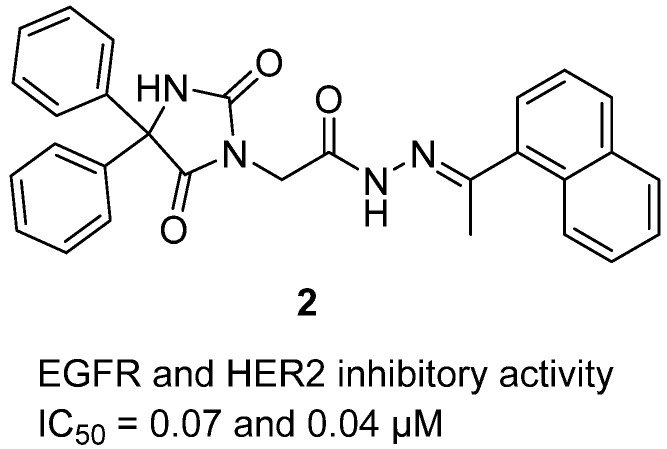
1-(Naphthalen-1-yl)ethylidene)hydrazide-tethered diphenylhydantoin **2**.

In 2025, synthesis of a library of Schiff’s bases incorporating the 5,5-diphenylhydantoin scaffold and evaluation for antiproliferative and kinase inhibitory activity were reported [23]. Among these, hydantoin **2**, bearing a 1-(naphthalen-1-yl)ethylidene)hydrazide substituent, emerged as a lead candidate (Figure 3). Structurally, it combines the hydantoin nucleus with a hydrazide–hydrazone linker that introduces an extended aromatic system, favoring π–π stacking and hydrogen bonding within kinase ATP-binding sites. Biologically, hydantoin **2** displayed potent inhibition of EGFR and HER2 tyrosine kinases with IC_50_ values of 0.07 µM and 0.04 µM, respectively, comparable to erlotinib (EGFR, 0.05 µM) and lapatinib (HER2, 0.03 µM). In cytotoxicity assays, it suppressed the growth of MCF-7 breast cancer cells (IC_50_ = 4.92 µM), HCT-116 colorectal carcinoma (12.83 µM), and HePG-2 hepatoblastoma cells (9.07 µM), while showing lower toxicity toward normal WI-38 fibroblasts (IC_50_ = 39.5 µM), translating into favorable selectivity indices. Mechanistic evaluation confirmed that compound 24 downregulated anti-apoptotic proteins BCL-2 and MCL-1 and induced cell cycle arrest at the G2/M phase, consistent with apoptosis induction. Molecular docking and 100 ns molecular dynamics simulations further validated its stable binding at the hinge regions of both EGFR and HER2 kinases, showing interaction networks comparable to co-crystallized inhibitors (gefitinib, Pro-03Q).

**Figure 4:**
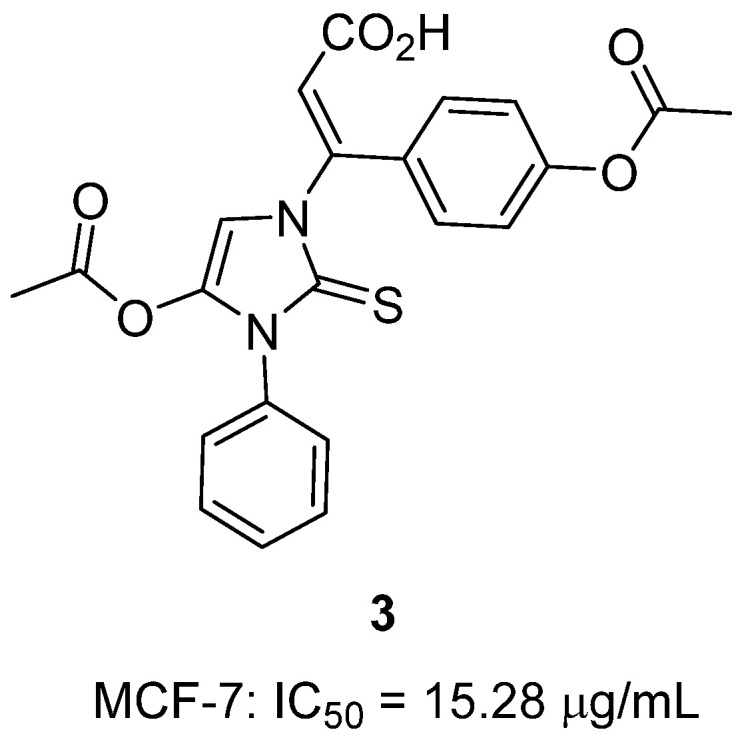
4-acetoxy-2-thioxo-imidazole as a derivative of 2-thiohydantoin **3**.

In 2024, Binjawhar and colleagues published the synthesis of hybrid cinnamic acid derivatives incorporating a 2-thiohydantoin scaffold as potential anti-breast cancer agents [24]. Within this series, 2-thiohydanoin **3**, carrying an acetoxy-substituted cinnamic acid moiety tethered to a thiohydantoin nucleus, was identified as the most active analogue (Figure 4). In vitro assays demonstrated that it inhibited MCF-7 breast cancer cell proliferation with an IC_50_ of 15.28 µg/mL, approaching the potency of doxorubicin (11.8 µg/mL) used as a positive control. Mechanistic studies revealed that compound **3** caused cell cycle arrest at the S phase (increasing S-phase cell population from 29% to 38%) and strongly induced apoptosis, with flow cytometry showing a marked rise in both early and late apoptotic cell populations relative to untreated controls. At the molecular level, qRT-PCR analyses showed that 2-thiohydanoin **3** downregulated Nrf2 and topoisomerase II expression while upregulating caspase-9, a key initiator of the intrinsic apoptotic pathway, thereby linking its cytotoxicity to both oxidative stress sensitization and DNA damage. Complementary molecular docking confirmed stable binding interactions with Nrf2, topoisomerase IIα/β, and caspase-9, consistent with its observed dual-target effects. Taken together, these results highlight 2-thiohydanoin **3** as a promising hybrid thiohydantoin–cinnamic acid anticancer lead, functioning through multi-target modulation of cell cycle progression, DNA repair enzymes, and redox regulation, and offering a rational framework for future optimization toward breast cancer therapy.

**Figure 5:**
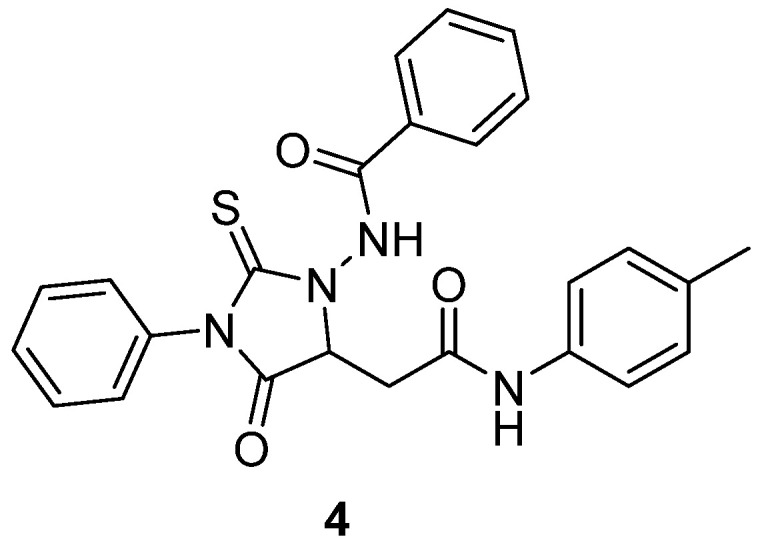
*N*-(2-thiohydatoinyl)benzamide **4**.

In 2023, Al-Shawi and co-workers investigated the anticancer mechanism of a novel 2-thiohydantoin derivative, N-(4-oxo-5-(2-oxo-2-(p-tolylamino)ethyl)-3-phenyl-2-thioxoimidazolidin-1-yl)benzamide (**4**) [25] (Figure 5). This compound was synthesized and evaluated for its effects on liver hepatocellular carcinoma (LIHC), particularly in HepG2 cells, where it exhibited potent cytotoxicity with an IC_50_ of 2.448 µM. Flow cytometry showed that thiohydanoin **4** induced cell cycle arrest in the S phase, reduced reactive oxygen species (ROS) production, and significantly increased late apoptosis relative to controls, suggesting a multi-pronged mode of action. Molecular docking and molecular dynamics simulations revealed high-affinity interactions with AKT1 (−10.4 kcal/mol) and CDK2 (−9.6 kcal/mol), two kinases implicated in proliferation and survival signaling. Bioinformatics analyses further linked high expression of AKT1 and CDK2 to poor prognosis in LIHC patients and showed their correlation with immune cell infiltration patterns—positively with macrophages, neutrophils, and eosinophils, and negatively with CD8^+^ T cells—suggesting immunomodulatory relevance. These results establish thiohydantoin **4** as a promising anticancer lead, functioning through inhibition of AKT1 and CDK2, modulation of oxidative stress, cell cycle blockade, and apoptosis induction, making it a strong candidate scaffold for further development in liver cancer therapeutics.

**Figure 6:**
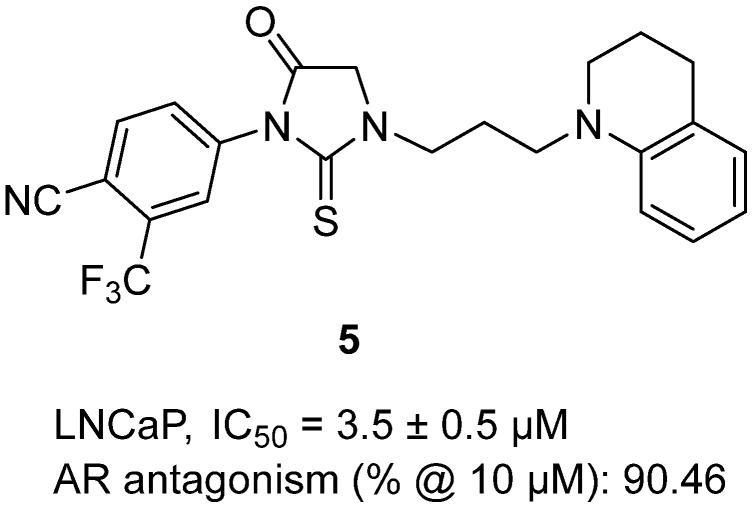
1-*N*-alkyl-2-*N*-arylthiohydanoin as an AR inhibitor and degrader.

In 2023, Chang and colleagues described the development of novel thiohydantoin-based androgen receptor (AR) antagonists designed not only to block AR activity but also to induce AR degradation, aiming to overcome resistance in prostate cancer [26]. Within this series, thiohydantoin **5**, featuring a trifluoromethyl–cyano phenyl group linked to a thiohydantoin core and extended with a tetrahydroquinoline moiety, stood out as a potent lead (Figure 6). In vitro, **5** inhibited LNCaP prostate cancer cell proliferation with an IC_50_ of 3.5 ± 0.5 µM, significantly outperforming enzalutamide (IC_50_ = 13.9 µM), while remaining inactive in AR-independent DU145 cells, confirming target selectivity. At 10 µM, 26 h exhibited robust AR antagonism (90.46% inhibition) and further demonstrated the ability to degrade both full-length AR and the constitutively active splice variant AR-V7 via a ubiquitin–proteasome-dependent pathway. Mechanistic assays showed that 26 h blocked AR nuclear translocation, disrupted AR/AR-V7 heterodimerization, suppressed downstream transcription (KLK3, FKBP5), and induced G1/M cell cycle arrest. In vivo, intraperitoneal dosing of **5** (30 mg/kg) achieved strong tumor growth inhibition in LNCaP (TGI = 70.7%) and 22Rv1 (TGI = 78.9%) xenograft models, outperforming enzalutamide under the same conditions, with no observable systemic toxicity. Overall, these findings establish **5** as a dual-function AR antagonist and degrader, highlighting the thiohydantoin scaffold as a valuable platform for next-generation prostate cancer therapy.

**Figure 7:**
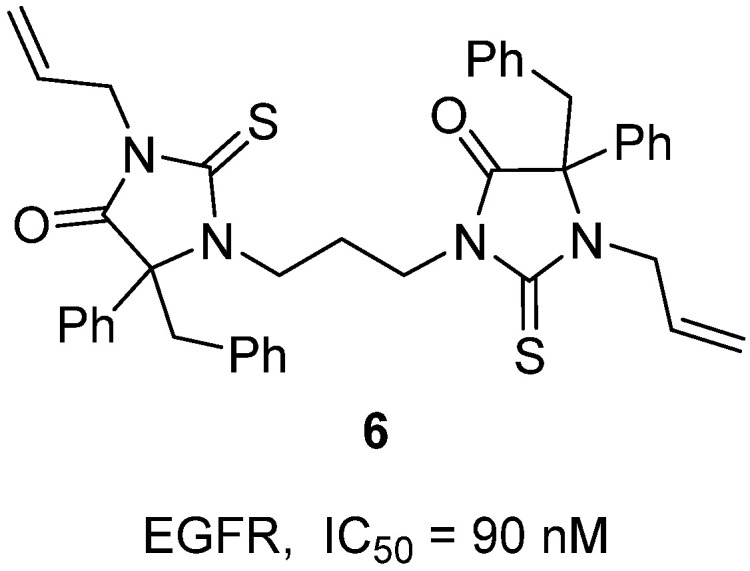
Dimeric thiohydantoin **6** as an EGFR tyrosine kinase inhibitor.

In 2024, Hassan and colleagues reported the synthesis of a novel series of bis-thiohydantoin derivatives as antiproliferative agents targeting the EGFR signaling pathway [27]. These compounds were prepared by reacting *N*,*N*″-(1,ω-alkanediyl)bis(*N*′-organylthiourea) derivatives with 2,3-diphenylcyclopropenone, affording symmetrical bis-thiohydantoins confirmed by NMR, MS, and by single-crystal X-ray analysis. Within this series, bisthiohydantoin **6**, containing an allyl substituent and a three-carbon linker, emerged as the most potent analogue (Figure 7). Biologically, **6** demonstrated strong antiproliferative activity across four human cancer cell lines (MCF-7, A549, Panc-1, and HT-29) with a mean GI_50_ of 1.20 µM, nearly equivalent to doxorubicin (GI_50_ = 1.10 µM). In enzymatic assays, **6** inhibited EGFR tyrosine kinase with an IC_50_ of 90 nM, closely approaching the potency of erlotinib (IC_50_ = 70 nM). Structure–activity relationship (SAR) analysis revealed that elongating the linker from two carbons to three carbons significantly improved potency, while substituent variation at the thiohydantoin N-3 position modulated activity (allyl > phenyl > ethyl). Molecular docking studies further supported these findings, showing that **6** achieved the highest binding affinity within the EGFR active site, stabilized by hydrogen bonds with Ser696 and Phe699, in a manner similar to erlotinib. These results identify bis-thiohydantoin scaffold **6** as a promising EGFR-targeted anticancer lead, meriting further structural optimization for clinical development.

**Figure 8:**
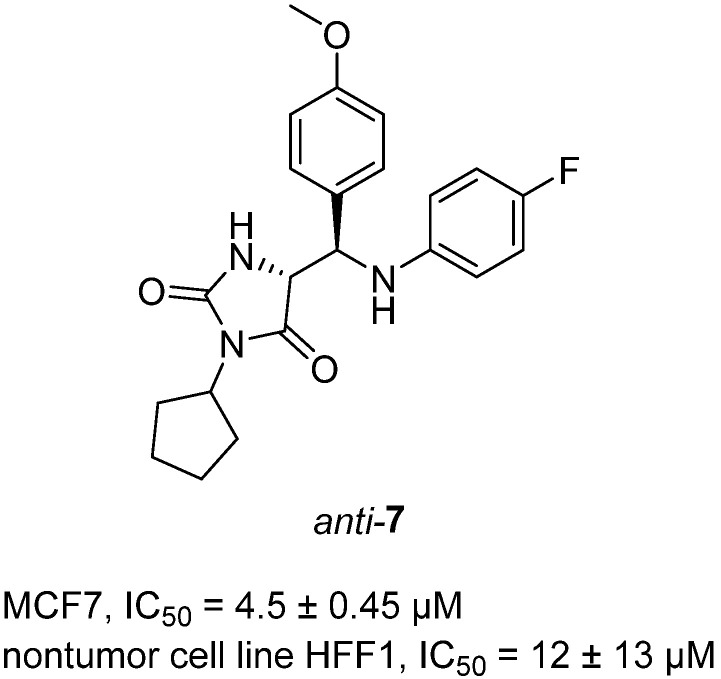
Chiral 3,5-disubstitute hydantoin **7** (*anti*-**7**).

In 2024, Jurin et al. reported the synthesis and biological profiling of a library of 3,5-disubstituted hydantoins prepared via base-assisted intramolecular amidolysis of β-lactam precursors [28]. The compounds were isolated as racemic mixtures of syn- and anti-isomers, with selected enantiomers characterized using electronic and vibrational circular dichroism (ECD/VCD) combined with DFT calculations to assign absolute configurations. Among the panel, compound hydantoin *anti*-**7**, bearing a cyclopentyl group at the N-3 position, displayed the most notable activity. It inhibited proliferation of MCF-7 breast adenocarcinoma cells with an IC_50_ of 4.5 µM, while showing lower toxicity toward the non-tumor human fibroblast cell line HFF-1 (IC_50_ = 12.0 µM), indicating a measure of selectivity for tumor cells (Figure 8). In contrast, its diastereomer *syn*-**7** was much less active (IC_50_ = 41 µM against MCF-7), underscoring the critical influence of stereochemistry on activity. Compound *anti*-**7** also showed moderate activity against A2780 ovarian carcinoma cells, but was largely inactive against HepG2 hepatocellular carcinoma cells. Overall, this work established that 3,5-disubstituted hydantoins are drug-like, membrane-permeable, and metabolically stable small molecules, with *anti*-**7** emerging as a promising lead for further optimization in anticancer drug discovery. The structure–activity relationship (SAR) analysis of the 3,5-disubstituted hydantoins highlights how the nature and size of the N-3 substituent strongly influence antiproliferative potency. The cyclopentyl-substituted *anti*-**7** was the most potent member of the series, showing IC_50_ = 4.5 µM against MCF-7 breast adenocarcinoma cells, with reasonable selectivity over the non-tumor fibroblast HFF-1 line (IC_50_ = 12.0 µM). In contrast, the linear hexyl analogue showed only moderate activity across tumor lines, with IC_50_ values around 30–35 µM, suggesting that excessive chain flexibility reduces productive interactions at the cellular target(s). Interestingly, the bulky aromatic tert-butylphenyl derivatives retained moderate potency in multiple cell lines (IC_50_ = 15–39 µM), consistent with the idea that steric bulk at N-3 can be tolerated but not optimized for tight binding. Taken together, the SAR trend suggests that a compact, conformationally restricted hydrophobic group such as cyclopentyl at N-3 maximizes cytotoxic potency and selectivity, whereas overly flexible (hexyl) or overly bulky/aromatic (t-BuPh) substituents compromise activity. These findings position *anti*-**7** as the lead analogue, and indicate that fine-tuning steric bulk and polarity at N-3 is a promising strategy for further optimization of this hydantoin scaffold.

**Figure 9:**
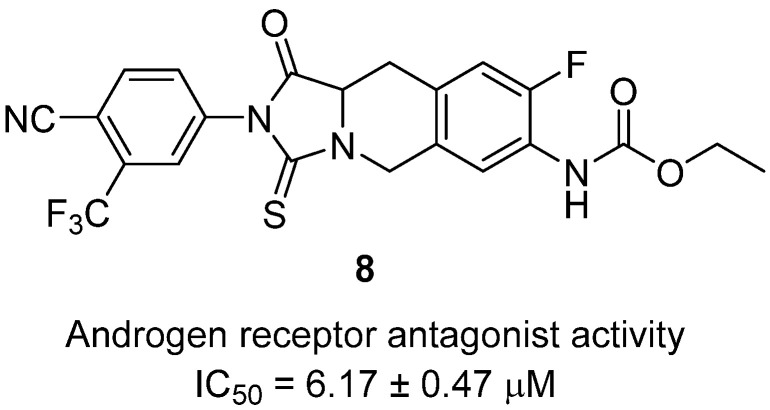
Tetrahydroisoquinoline-fused hydantoin **8**.

Xu and co-workers reported the discovery of pyridine tetrahydroisoquinoline thiohydantoin derivatives as a new class of androgen receptor (AR) antagonists with reduced blood–brain barrier penetration, aiming to improve safety over current antiandrogens such as enzalutamide [29]. Within this series, hydantoin **8** was identified as a lead in 2020 (Figure 9). Structurally, it integrates a pyridine-linked tetrahydroisoquinoline core fused to a thiohydantoin ring with a fluorine substituent and an ethyl carbamate side chain, rationally designed to provide AR binding affinity while limiting CNS penetration. Biologically, **8** exhibited potent AR antagonism with an IC_50_ of 6.17 ± 0.47 µM, close to enzalutamide, and showed strong inhibition of AR-dependent LNCaP prostate cancer cells (IC_50_ = 14.8 µM), while remaining essentially inactive on AR-independent DU145 cells (IC_50_ > 100 µM), confirming selectivity for AR-driven proliferation. Importantly, **8** effectively blocked AR nuclear translocation in immunofluorescence assays and inhibited AR-mediated gene transcription. In vivo, **8** significantly suppressed tumor growth in LNCaP xenograft models with a tumor growth inhibition rate of 79% at 45 mg/kg, outperforming enzalutamide (68%), and produced a 73% reduction in serum PSA, without apparent systemic toxicity. Pharmacokinetic studies demonstrated low brain penetration relative to plasma levels, suggesting a reduced seizurogenic risk compared to enzalutamide. These findings highlight **8** as a promising AR antagonist scaffold that balances efficacy, safety, and pharmacokinetic advantages, making it a valuable lead for next-generation prostate cancer therapies.

**Figure 10:**
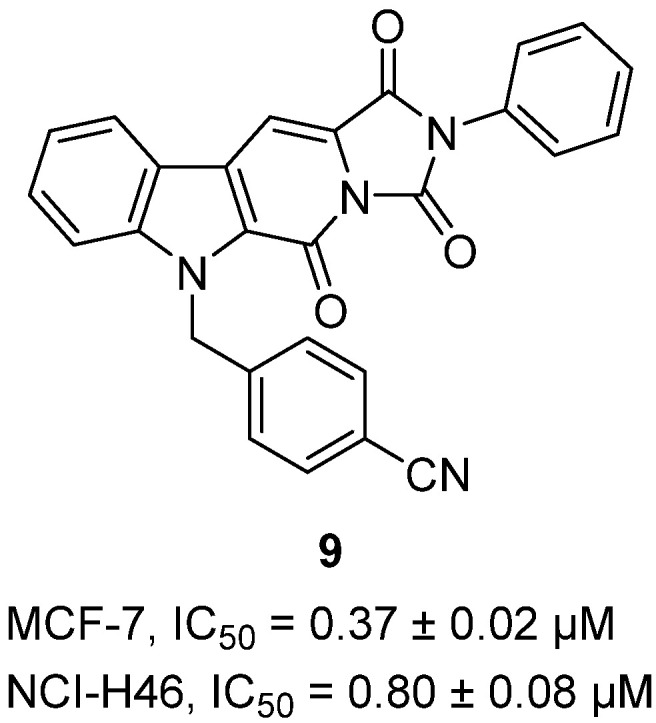
β-Carbolinone-fused hydantoin **9**.

In 2020, Yao and co-workers designed and synthesized a series of β-carboline-1-one hydantoins as rigidified aplysinopsin-inspired scaffolds using a tandem Knoevenagel condensation–intramolecular cyclization strategy [30]. The tetracyclic framework was selected to impose conformational restriction and enhance anticancer potency. Among the library, hydantoin **9**, bearing a para-cyano-substituted benzyl group at the N-1 position, was identified as the most active derivative (Figure 10). Biologically, it exhibited strong cytotoxicity against multiple human cancer cell lines: in MCF-7 breast cancer cells, it inhibited growth by 59% at 10 µM with an IC_50_ of 0.37 ± 0.02 µM, making it 83-fold more potent than the clinical standard 5-fluorouracil (IC_50_ = 30.76 µM). In NCI-H460 lung cancer cells, it showed 87% inhibition at 10 µM and an IC_50_ of 0.80 ± 0.08 µM, again outperforming 5-FU (IC_50_ = 1.54 µM). These results demonstrate that the β-carboline-1-one hydantoin scaffold is a privileged structural motif for anticancer activity, with **9** representing a promising lead compound for further development. Its performance across breast and lung models underscores the potential of this scaffold for advancing novel small-molecule therapeutics targeting solid tumors.

**Figure 11:**
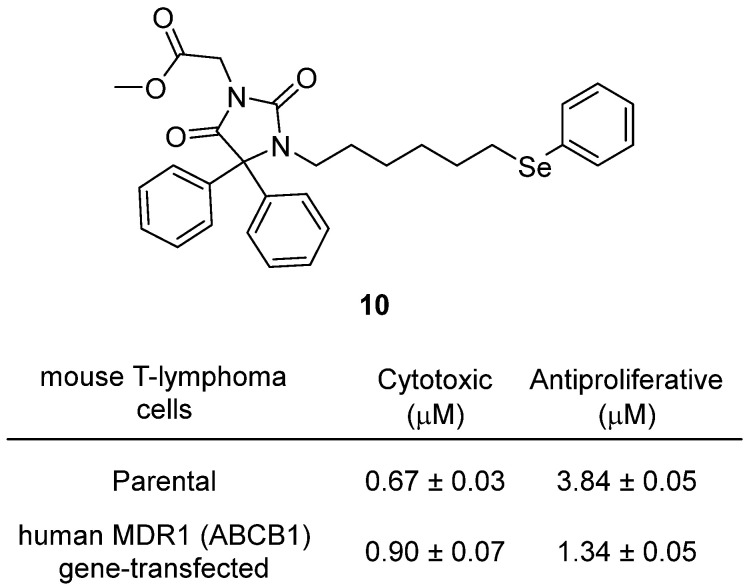
Hydantoin–selenoether hybrid **10**.

Ali and colleagues reported the discovery of phenylselenoether–hydantoin hybrids as modulators of the ABCB1 (P-glycoprotein/MDR1) efflux pump with notable cytotoxic and antiproliferative activity in resistant T-lymphoma cells in 2020 [31]. Among these, hydantoin **10**, a 5,5-diphenylhydantoin derivative bearing a phenylselenoether chain, emerged as the most active (Figure 11). Structurally, the combination of a hydantoin scaffold—well known for pharmacological versatility—with a phenylselenoether linker endowed the molecule with both ABCB1 modulation and intrinsic cytotoxicity. In human MDR1 gene–transfected mouse lymphoma cells, **10** strongly inhibited proliferation with IC_50_ values of 0.67 ± 0.03 µM (parental cells) and 0.90 ± 0.07 µM (MDR cells), whereas resistant cells typically show decreased sensitivity. Antiproliferative effects were also pronounced, with IC_50_ = 3.84 µM in parental cells vs. 1.34 µM in MDR cells, highlighting enhanced activity in resistant phenotypes, unlike many conventional agents. Mechanistic studies showed that hydantoin–selenoether hybrids such as **10** act as ABCB1 substrates, competitively displacing rhodamine-123 in efflux as-says and stimulating Pgp ATPase activity, suggesting that efflux modulation occurs via substrate competition. Moreover, in human JURKAT T-lymphoblastic leukemia cells, **10** reduced cell proliferation, downregulated cyclin D1, and upregulated p53, thereby potentiating apoptosis. When combined with doxorubicin, it exhibited synergistic antiproliferative effects, restoring chemosensitivity in resistant cells. Overall, **10** represents a promising lead, demonstrating that hydantoin–selenoether hybrids can simultaneously act as efflux pump modula-tors and cytotoxic agents, providing a novel chemotype for overcoming multidrug resistance in cancer.

**Figure 12:**
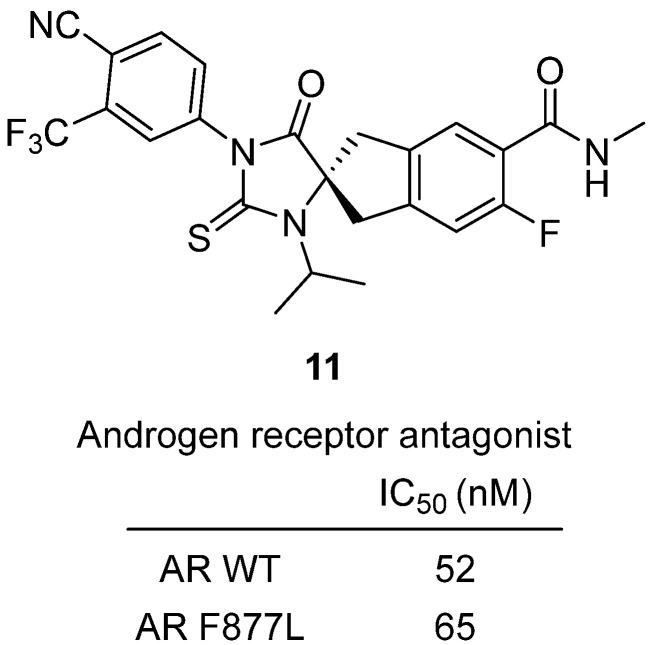
Spirocyclic thiohydantoin **11**.

In 2021, a study by Zhang and colleagues was reported the discovery of spirocyclic thiohydantoin antagonists targeting both wild-type AR and the resistance-conferring F877L mutant [32]. Castration-resistant prostate cancer (CRPC) frequently develops resistance to second-generation antiandrogens such as enzalutamide and apalutamide through AR ligand-binding domain mutations like F877L, which can switch antagonists into agonists. To overcome this, the team designed rigidified spirocyclic thiohydantoin scaffolds to restrict ligand flexibility and maintain antagonism. Among these, compound thiohydantoin **11** emerged as a standout, acting as a full antagonist in both AR WT and F877L reporter assays with IC_50_ values of 52 nM and 65 nM, respectively, and achieving >90% maximal inhibition (Figure 12). This potency exceeded enzalutamide, which fails against AR F877L. In androgen-dependent VCaP prostate cancer cells, **11** inhibited proliferation with an IC_50_ of ~40 nM, comparable to benchmark AR antagonists. Pharmacokinetic profiling in mice showed good oral bioavailability (80–90%) and favorable clearance, while in vivo studies demonstrated that **11** significantly reduced androgen-sensitive organ growth in the Hershberger assay and inhibited tumor progression in LNCaP F877L xenograft models. These results confirm that **11** is a potent, orally bioavailable, and selective AR antagonist that avoids the antagonist-to-agonist switch, underscoring the therapeutic promise of spirocyclic thiohydantoins as next-generation treatments for CRPC resistant to current antiandrogens.

**Figure 13:**
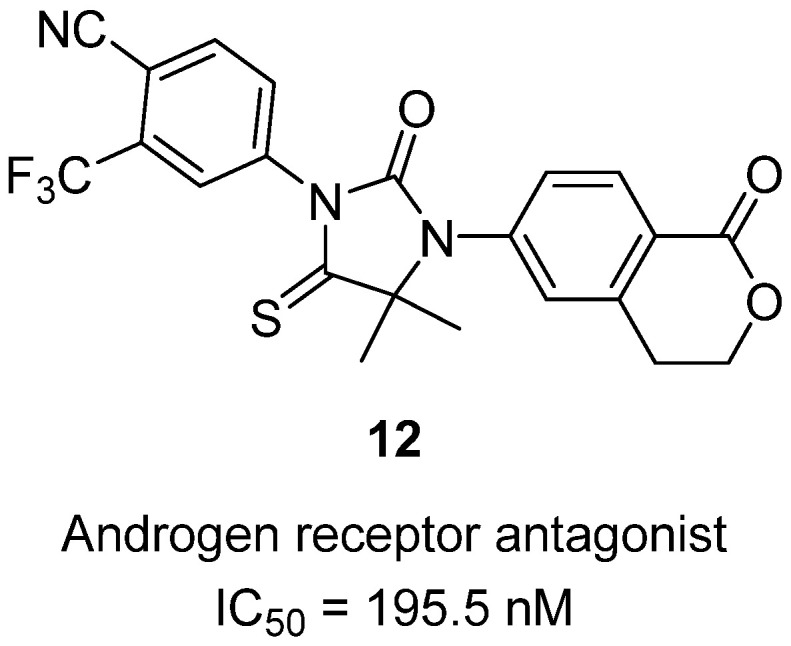
2-Thiohydantoin-isochromanone hybrid **12**.

In 2021, Wang and co-workers described the design of novel thiohydantoin derivatives as potent androgen receptor (AR) antagonists for prostate cancer therapy [33]. Prostate cancer, particularly in its castration-resistant form (CRPC), remains highly dependent on AR signaling, and resistance to current AR antagonists such as enzalutamide often emerges. To address this, the authors developed series 2-thiohydantoin-lactone hybrids, incorporating the thiohydantoin scaffold linked to an isobenzofuranone moiety, aiming to increase steric hindrance against AR helix-12 and enhance antagonist binding. Among the tested compounds, **12** stood out, showing AR antagonistic activity with an IC_50_ of 195.5 nM, which is stronger than enzalutamide (IC_50_ ≈ 454.6 nM) (Figure 13). In antiproliferative assays, **12** inhibited the growth of LNCaP prostate cancer cells (IC_50_ = 1.94 µM) and AR-overexpressing LNCaP/AR cells (IC_50_ = 0.73 µM), essentially matching the performance of enzalutamide. Molecular docking studies revealed that **12** adopts a binding mode similar to enzalutamide but forms additional hydrogen bonds with AR residues Arg752, Gln711, and Arg779, and π–π stacking with Phe764, providing a structural rationale for its enhanced potency. Thiohydantoin **12** demonstrates that thiohydantoin is a privileged scaffold for AR inhibition, offering both sub-200 nM antagonist potency and robust cell-based efficacy, highlighting its potential as a lead compound for further development against CRPC.

**Figure 14:**
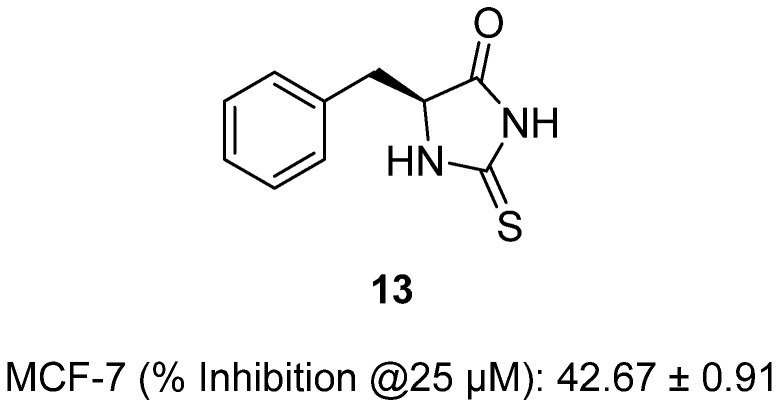
(*S*)-5-Benzyl-2-thiohydantoin **13**.

In 2022, Fagundes and colleagues investigated a series of thiohydantoin derivatives for activity against MCF-7 human breast cancer cells [34]. Among these, benzylthiohydantoin **13**, a phenyl-substituted thiohydantoin, inhibited MCF-7 cell growth by 42.67% at 25 µM, showing significantly greater activity than the reference drug doxorubicin under the same conditions (Figure 14). Structurally, thiohydantoins are sulfur analogues of hydantoins, where one carbonyl is replaced by a thioxo group, conferring lipophilicity and binding affinity to biomacromolecules. Biological evaluation demonstrated that active members such as **13** induced cytotoxicity via increased reactive oxygen/nitric oxide production, mitochondrial depolarization, and lysosomal dysfunction, leading to apoptosis and autophagy-associated cell death. Mechanistic assays confirmed caspase-3 activation, phosphatidylserine externalization, and G0/G1 cell cycle arrest, highlighting disruption of tumorigenic signaling and mitochondrial viability as central to the antitumor effect.

**Figure 15:**
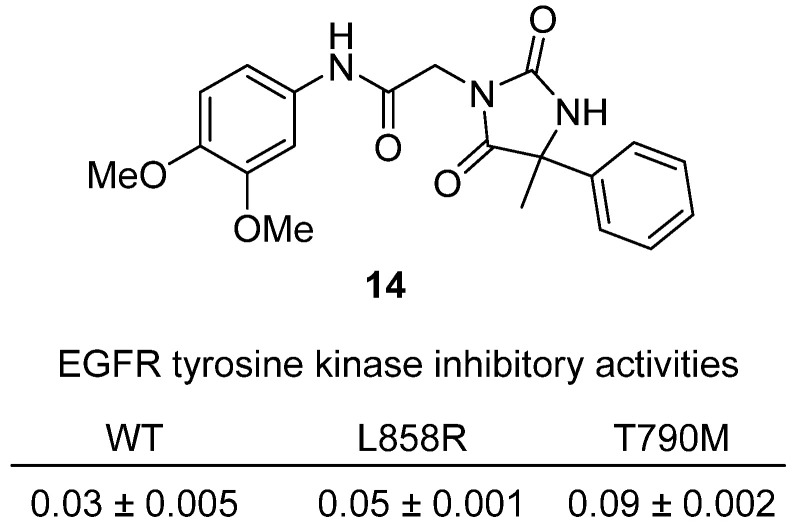
2-Hydantoinylacetamide **14**.

Hassanin and colleagues designed and synthesized a new series of hydantoin acetanilide derivatives to target EGFR wild type and the resistance mutations L858R/T790M in non-small cell lung cancer (NSCLC) in 2022 [35]. Among the set, **14** emerged as the most potent (Figure 15). Structurally, both adopt a 5-methyl-5-phenylhydantoin core N-alkylated via a chloroacetamide linker to differently substituted anilides; this arrangement was modeled to hydrogen-bond with Thr854 and Lys745, while the hydrophobic acetanilide engages Cys797, enabling interactions around Met790 in the resistant EGFR variant. Biochemically, **14** displayed IC_50_ values of 0.03 µM (WT), 0.05 µM (L858R), and 0.09 µM (T790M), essentially matching erlotinib on the mutantss. In antiproliferative tests, **14** inhibited A549, H1975, and PC9 cells with IC_50_ values of 2.26, 1.94, and 10.19 µM, respectively, outperforming erlotinib particularly against the resistant H1975 line. Mechanistic studies further confirmed that **14** strongly inhibited EGFR downstream signaling (Akt IC_50_ = 33.8 nM, ERK IC_50_ = 25.8 nM) and reduced EGFR phosphorylation in Western blot assays, while both compounds showed low cytotoxicity against normal WI-38 fibroblasts. These findings highlight hydantoin-based acetanilides as promising mutant-active EGFR inhibitors, with **14** representing a rationally designed hinge-binding lead and 5f demonstrating excellent translation into cellular efficacy.

**Figure 16:**
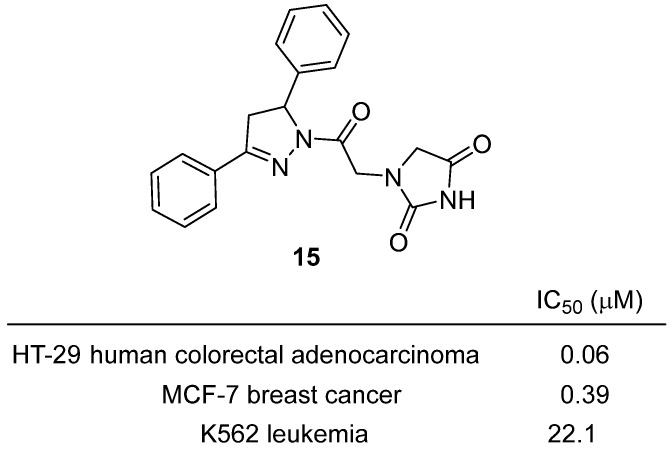
Pyrazoline-linkered hydantoin **15**.

In a 2021 study, Upadhyay and colleagues described the development of novel pyrazoline-based hydantoin analogs using a pharmacophore hybridization strategy, aiming to enhance anti-tumor efficacy and mechanistic diversity [36]. Within this series, hydantoin **15**, featuring an unsubstituted diphenylpyrazoline core linked to an imidazolidine-2,4-dione (hydantoin) ring, stood out as the most potent lead (Figure 16). **15** inhibited proliferation of HT-29 human colorectal adenocarcinoma cells with an IC_50_ of 10 nM, which was 6-fold more potent than paclitaxel (IC_50_ = 60 nM) and at least 10-fold more active than the corresponding pyrrolidine-2,5-dione hybrid. **15** also showed strong cytotoxicity against MCF-7 breast cancer cells (IC_50_ = 0.39 µM) and moderate activity in K562 leukemia cells (IC_50_ = 22.1 µM), indicating selectivity for solid tumor lines. Mechanistic assays revealed that **15** induced caspase activation, arrested the cell cycle at the G0/G1 phase, decreased anti-apoptotic Bcl-2 protein expression, and increased DNA double-strand breaks, supporting a multi-modal pro-apoptotic mechanism.

Molecular docking studies demonstrated that the (R)-enantiomer of **15** fits deeply into the hydrophobic groove of Bcl-2, forming hydrogen bonds and hydrophobic interactions with key residues, rationalizing its Bcl-2 inhibitory activity. In vivo, oral and intraperitoneal administration of **15** (50 mg/kg) in HT-29 xenograft mouse models resulted in significant tumor growth inhibition (TGI = 44% per os, 36% i.p.), with good oral bioavailability and no acute toxicity at doses up to 100 mg/kg.

**Figure 17:**
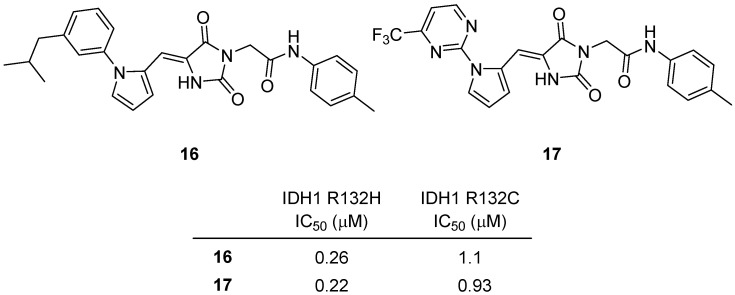
Pyrrolidenyl hydantoin **16** and **17**.

In 2025, Luo and colleagues reported the design, synthesis, and structure–activity relationship (SAR) optimization of novel hydantoin-based inhibitors targeting mutant isocitrate dehydrogenase 1 (mIDH1) enzymes, specifically the R132H and R132C variants [37]. Two hydantoins, **16** and **17**, emerged as the standout leads following extensive SAR exploration of over forty analogues across four structural domains (Figure 17). Both compounds acted as potent, selective inhibitors of mIDH1, effectively suppressing pathological 2-hydroxyglutarate (2-HG) accumulation in mutant cell lines. Compound **16** features an isobutyl–substituted phenyl moiety tethered to a hydantoin scaffold via a pyrrole linker and p-tolylacetamide chain. The SAR analysis indicated that replacing the trifluoromethyl group of the initial hit compound with an isobutyl group profoundly enhanced inhibitory potency, achieving complete inhibition of IDH1 R132H and R132C at 2 and 10 µM, respectively. **16** demonstrated an IC_50_ of 0.26 µM for IDH1 R132H and 1.1 µM for IDH1 R132C, while showing no measurable inhibition of wild-type IDH1/2 even at 40 µM, confirming high selectivity. In cellular assays using U87-MG R132H glioma cells, **16** reduced intracellular 2-HG production with an EC_50_ of 0.55 µM and exhibited moderate antiproliferative effects without cytotoxicity in normal human L02 hepatic cells. Pharmacokinetic evaluation further revealed favorable brain penetration (brain-to-plasma ratio ≈ 0.84) and an acceptable oral bioavailability profile, supporting its potential utility in treating IDH1-mutant gliomas. Compound **17**, incorporating a 2-pyrimidinyl substituent at the same position as the phenyl group in **16**, also displayed strong activity. It inhibited the IDH1 R132H enzyme with an IC_50_ of 0.22 µM and the R132C mutant with an IC_50_ of 0.93 µM. However, its intracellular activity (EC_50_ = 1.45 µM for 2-HG inhibition) was somewhat lower than **16**, likely due to reduced cell permeability. Both **16** and **17** shared a common mechanism of engaging the monomeric allosteric site of mIDH1, blocking the pathological NADPH-dependent reduction of α-ketoglutarate to 2-HG. Compounds **16** and **17** represent a pair of highly selective, brain-penetrant, hydantoin-based mIDH1 inhibitors with low toxicity and robust biochemical potency. Their complementary structural frameworks—hydrophobic isobutyl substitution (**16**) versus heteroaromatic pyrimidine incorporation (**17**)—define key pharmacophoric elements that will guide subsequent optimization toward clinical candidate development for mIDH1-driven malignancies.

**Figure 18:**
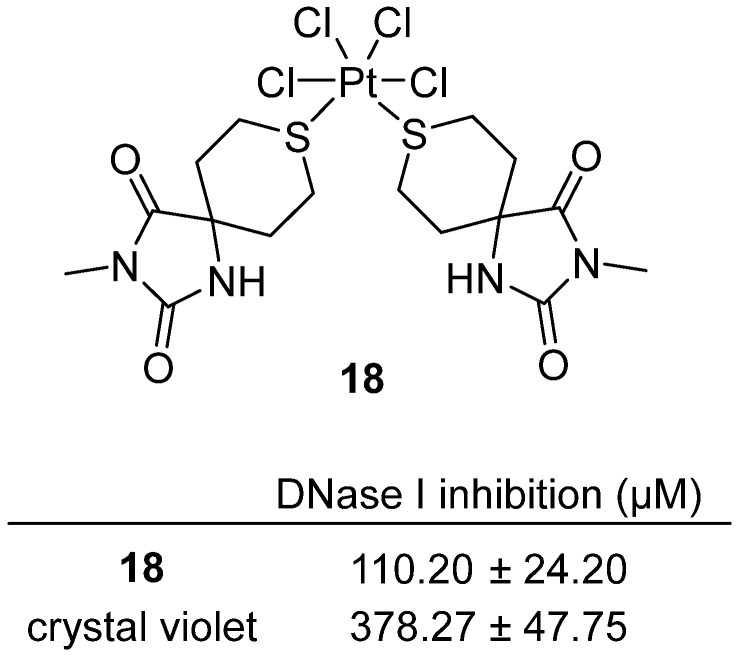
Hydantoin-containing platinum complex **18**.

Marković et al. reported the synthesis and biological evaluation of a series of hydantoin-containing metal complexes as potential deoxyribonuclease I (DNase I) inhibitors in 2024 [38]. Among these, compound **18**, a platinum(IV) complex of 3′-methyl-4-thio-1H-tetrahydropyranspiro-5′-hydantoin, emerged as the most potent DNase I inhibitor in the series (Figure 18). **18** inhibited bovine pancreatic DNase I with an IC_50_ of 110.2 ± 24.2 µM, demonstrating approximately threefold greater potency than the reference inhibitor crystal violet (IC_50_ = 378.3 ± 47.8 µM). Molecular docking studies revealed that **18** binds within the enzyme’s catalytic site, forming hydrogen bonds with key residues such as Asn7, Glu39, Ser110, and His134, which are critical for DNase I activity. This binding mode suggests that compound **18** acts as a competitive inhibitor, directly blocking the substrate’s access to the active site. Notably, this is the first report of a platinum complex exhibiting DNase I inhibitory activity.

**Figure 19:**
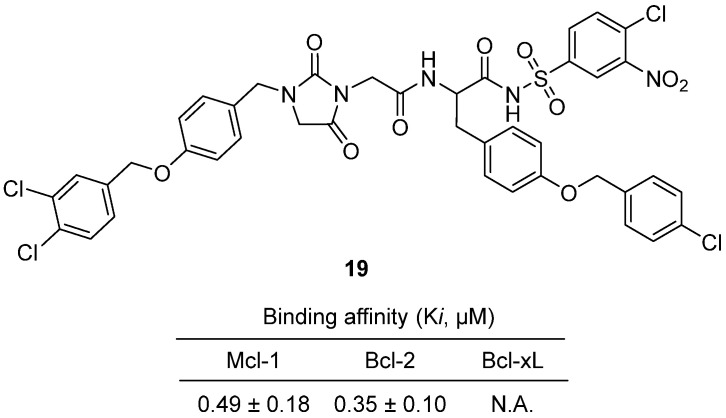
*N*,*N*’-Disubstituted hydantoin **19**.

In 2022, Liang and colleagues reported the design, synthesis, and biological evaluation of a series of hydantoin derivatives as selective Mcl-1 inhibitors for cancer therapy [39]. Among these, hydantoin **19**, featuring a 3,4-dichlorobenzyl group at the hydantoin core and a 4-chlorobenzyl-oxyphenyl motif, emerged as a potent lead (Figure 19). In vitro, **19** exhibited strong binding affinity to Mcl-1 (Ki = 0.49 μM), comparable to the reference inhibitor UMI-77, and showed good selectivity over Bcl-xL and moderate selectivity over Bcl-2, indicating reduced risk of platelet toxicity. **19** effectively inhibited proliferation of various cancer cell lines, including H929, THP-1, Jurkat, HCT116, HepG2, and PC-3, with IC_50_ values ranging from 10.12 to 17.82 μM, and demonstrated lower toxicity to normal cells compared to dual Bcl-2/Bcl-xL inhibitors. Mechanistic assays revealed that **19** induced apoptosis in HepG2 cells, as confirmed by TUNEL staining and annexin-V/7-AAD flow cytometry, and disrupted mitochondrial membrane potential, supporting its role in activating the intrinsic apoptotic pathway. Additionally, **19** showed good plasma stability, with approximately 80% remaining after 24 h in rat plasma at 37 °C, suggesting favorable pharmacokinetic properties. Structure–activity relationship (SAR) analysis highlighted that bulky, halogenated aromatic substitutions at the hydantoin core and side chains enhance Mcl-1 binding and selectivity.

### 2.2. Cardiovascular

**Figure 20:**
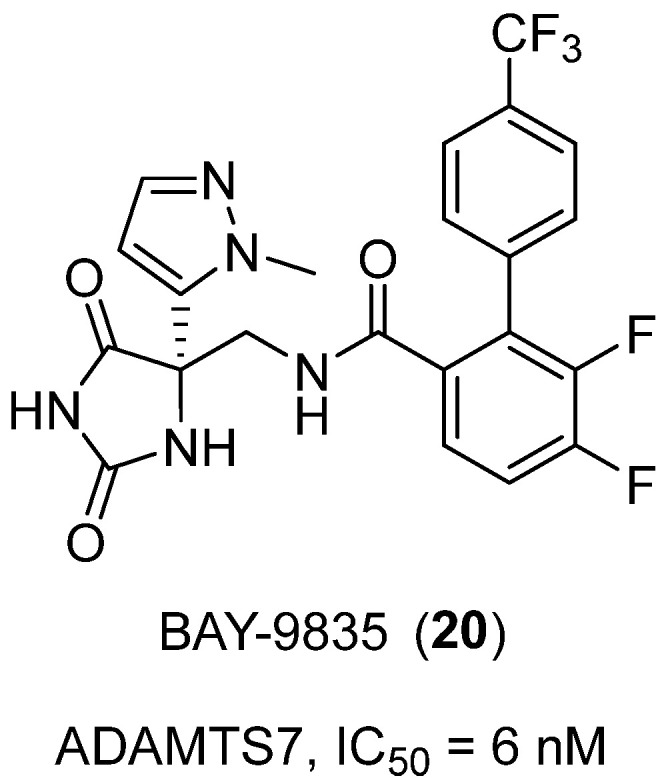
Structure of BAY-9835 (**20**).

BAY-9835 (**20**) is the first reported orally bioavailable small-molecule inhibitor of ADAMTS7, a zinc metalloprotease strongly implicated in atherosclerosis and restenosis [40]. Genome-wide association studies identified ADAMTS7 variants as risk factors for coronary artery disease (CAD), and functional studies showed that elevated ADAMTS7 activity promotes vascular smooth muscle cell (VSMC) and endothelial cell migration, contributing to neointimal hyperplasia and atherosclerotic plaque progression. Conversely, ADAMTS7 knockout in mice reduces atherosclerosis and vascular remodeling after injury. High ADAMTS7 expression has also been observed in human atherosclerotic lesions and injured vessels, correlating with worse cardiovascular outcomes. **20** was optimized from hydantoin-based scaffolds through structure-guided design to improve potency, selectivity, and pharmacokinetics, achieving an IC_50_ of 6 nM against ADAMTS7 (Figure 20). It shows high selectivity across metalloproteases, though it also inhibits ADAMTS12, whose role in pathology is more complex (sometimes protective, sometimes deleterious depending on context, e.g., in arthritis, cancer, or inflammation). In preclinical studies, **20** demonstrated low clearance, high oral bioavailability, good solubility, and tolerability in rats, without genotoxicity or off-target liabilities. This places **20** as a promising lead for therapeutic development targeting coronary artery disease, restenosis after vascular injury, and potentially other ADAMTS7-driven cardiovascular conditions.

**Figure 21:**
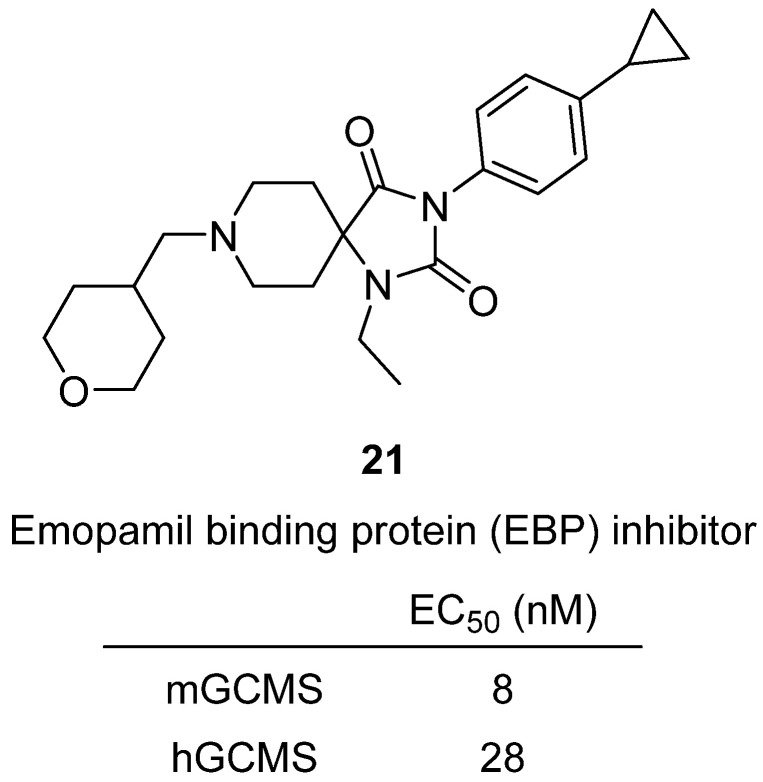
Spiro-fused piperidinohydantoin **21**.

In 2024, Dorel et al. at Genentech and Convelo Therapeutics reported the discovery and optimization of hydantoin-based inhibitors of emopamil binding protein (EBP), a sterol isomerase in the cholesterol biosynthesis pathway [41]. EBP inhibition causes the accumulation of 8,9-unsaturated sterols (e.g., zymostenol), which act as signaling lipids that enhance oligodendrocyte precursor cell (OPC) differentiation into myelinating oligodendrocytes—a potential regenerative approach for multiple sclerosis (MS) and other demyelinating diseases. The optimization campaign began from a spiroindolinone hit that promoted OPC differentiation but suffered from metabolic instability and off-target promiscuity. Cryo-EM structures of EBP with this ligand guided rational design, leading to a hydantoin scaffold that improved polarity, safety, and brain penetration while retaining high potency. Among the optimized analogues, **21** achieved an mGCMS EC_50_ of 8 nM and an hGCMS EC_50_ of 28 nM, with clean off-target and safety pharmacology profiles (Figure 21). It demonstrated good oral bioavailability, robust brain penetration (Kp,uu ~0.7–0.8 in rodents), and dose-dependent accumulation of zymostenol in the brain, confirming target engagement. Overall, this work establishes hydantoin-based EBP inhibitors such as **21** as first-in-class, brain-penetrant chemical probes for regenerative therapy, highlighting their promise as novel small-molecule approaches to promote remyelination in MS and related myelin disorders.

**Figure 22:**
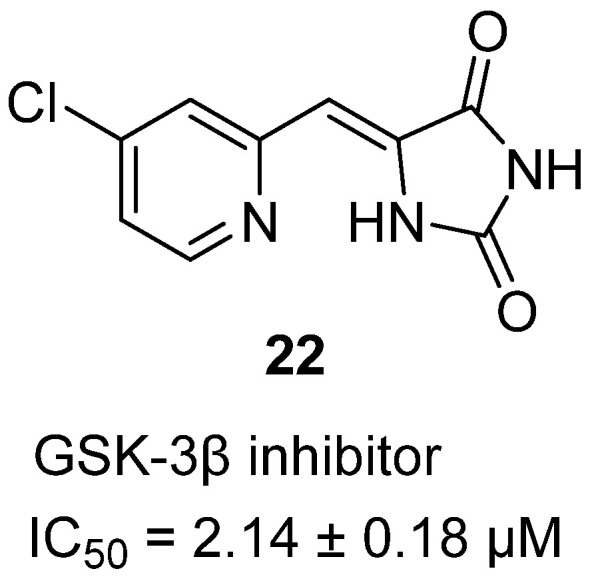
Pyridinylmethylenehydantoin **22** as GSK-3β inhibitor.

In 2024, Schneider and coworkers synthesized a family of 5-(heteroarylmethylene)hydantoins and evaluated them as glycogen synthase kinase-3β (GSK-3β) inhibitors, a kinase implicated in diverse pathologies such as diabetes, Alzheimer’s disease, cancer, and mood disorders [42]. Among the series, (*Z*)-5-[(6′-methyl-2-pyridinyl)methylene]hydantoin (**22**) stood out as one of the most potent inhibitors (Figure 22). Structurally, the compound features a hydantoin core conjugated with a substituted pyridyl ring, allowing potential intramolecular hydrogen bonding between the hydantoin N–H and the pyridine nitrogen, which imparts conformational bias and favors productive kinase binding. Biologically, **22** inhibited GSK-3β with an IC_50_ of 2.14 ± 0.18 µM, placing it among the most active analogues in the set. Selectivity profiling indicated that these hydantoin analogues exhibited minimal inhibition of off-target metalloenzymes such as MMP-12, carbonic anhydrase II, and *Staphylococcus aureus* pyruvate carboxylase, confirming a degree of kinase specificity. The results establish **22** as a promising micromolar GSK-3β inhibitor, reinforcing the hydantoin scaffold’s versatility as a kinase-targeting chemotype and laying the groundwork for further optimization toward potency and selectivity.

### 2.3. Antimicrobial

**Figure 23:**
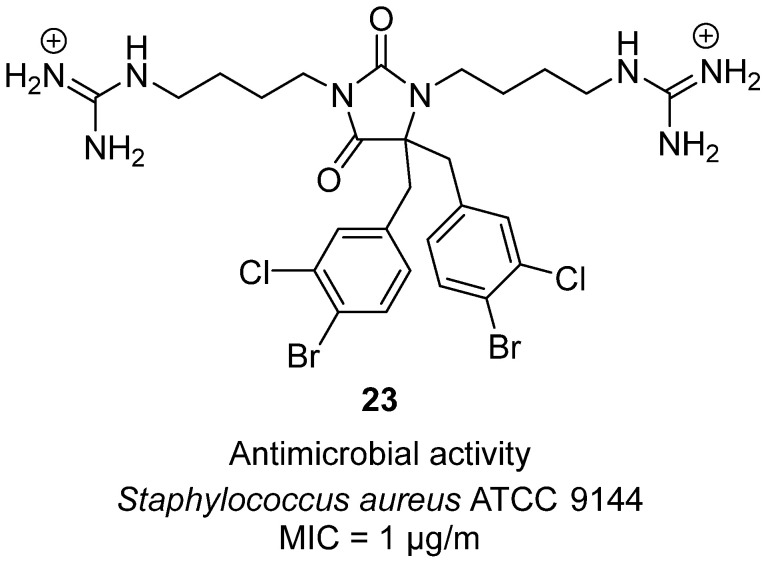
Cationic guanidine-tethered hydantoin **23**.

In 2023, Bayer et al. explored tetrasubstituted hydantoins as antimicrobial peptide mimics with membranolytic activity [43]. Their design strategy placed two lipophilic substituents (3,5-dibromophenyl) and two cationic polyamine/guanidine chains onto the hydantoin core, generating amphipathic molecules intended to disrupt bacterial membranes. Among the synthesized compounds, hydantoin **23** demonstrated outstanding potency, exhibiting a minimum inhibitory concentration (MIC) of 1 µg/mL against *Staphylococcus aureus* ATCC 9144 (Figure 23). Structurally, **23** is a hydantoin derivative bearing bulky dibromo-dichlorophenyl lipophilic groups and terminal polycationic guanidinium groups, which together mimic the amphipathic profile of natural antimicrobial peptides. Biological evaluation revealed that this scaffold provided potent Gram-positive antibacterial activity while maintaining very low hemolytic toxicity toward human red blood cells, a crucial balance for therapeutic viability. Mechanistic assays with luciferase-based biosensors confirmed that these hydantoins act primarily by rapid disruption of bacterial membranes in a concentration-dependent manner, akin to cationic antimicrobial peptides but with greater synthetic stability.

**Figure 24:**
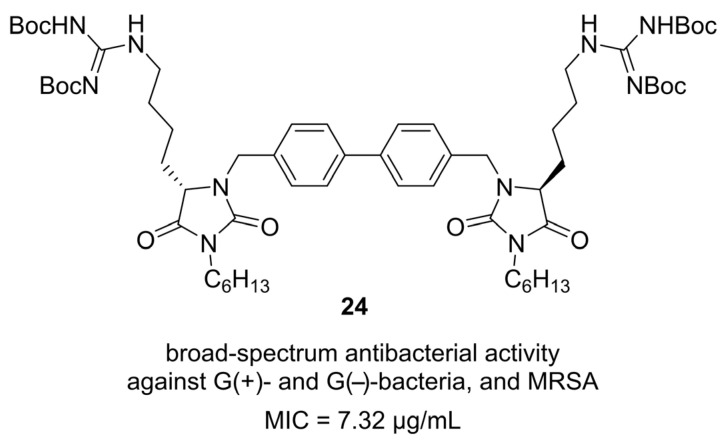
Biphenyl linkered dimeric hydantoin **24**.

In 2024, Chen and co-workers reported the design and evaluation of hydantoin derivative dimers as broad-spectrum antimicrobial agents against drug-resistant ESKAPE pathogens [44]. Inspired by antimicrobial peptides (AMPs), the researchers constructed amphiphilic dimers containing two hydantoin backbones, a rigid aromatic linker (e.g., biphenyl), long hydrophobic lipid tails, and cationic residues (lysine or arginine mimics). This arrangement generated charge-dispersed, membrane-active molecules with improved pharmacological properties over monomeric hydantoins. Among the library, hydantoin **24** emerged as the lead, showing potent broad-spectrum activity with a geometric mean MIC of 7.32 µg/mL against Gram-positive and Gram-negative bacteria, including MRSA (Figure 24). Mechanistic studies revealed that **24** rapidly killed both *S. aureus* and *E. coli* within 1 h at 1–4× MIC, disrupted both inner and outer bacterial membranes, increased intracellular ROS, and interacted with lipopolysaccharide (LPS) in Gram-negative bacteria. Notably, it maintained antibacterial efficacy over 25 serial passages, unlike ciprofloxacin and gentamicin which rapidly induced resistance. Additional assays confirmed that **24** possessed excellent stability under heat, salt, serum, and culture medium conditions, and scanning electron microscopy demonstrated membrane collapse and leakage in treated cells.

**Figure 25:**
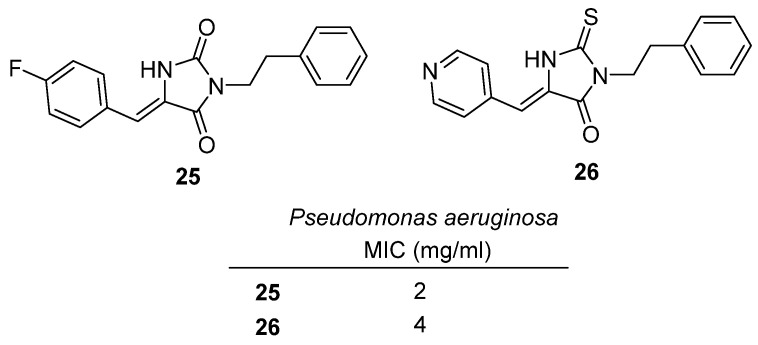
Arylidenehydantoin **25** and Arylidenethiohydantoin **26**.

In 2021, Mohamed and co-workers synthesized a library of imidazolidine-2,4-dione (hydantoin) and 2-thioxoimidazolidin-4-one (thiohydantoin) derivatives and evaluated them as inhibitors of virulence factor production in *Pseudomonas aeruginosa* [45]. The design aimed to interfere with quorum sensing (QS) regulators LasR and RhlR, thereby suppressing bacterial pathogenicity without directly affecting viability, an attractive antivirulence strategy against resistant *Pseudomonas* infections. Among the new derivatives, hydantoin **25** and thiohydantoin **26** were highlighted (Figure 25). Both displayed moderate antimicrobial activity, with MIC values of 2 mg/mL (**25**) and 4 mg/mL (**26**) against *P. aeruginosa*. Beyond growth inhibition, their more notable effects were on virulence suppression: **25** showed complete inhibition of protease and hemolysin production at sub-MIC levels (^1/4^ MIC), while **26** strongly inhibited pyocyanin production (96.4% at 1 mg/mL), a key toxin linked to oxidative stress and host tissue damage. These results demonstrate that hydantoin and thiohydantoin scaffolds can be tuned to selectively block different virulence pathways in *P. aeruginosa*, with **25** more effective on protease/hemolysin and **26** on pyocyanin. This work positions hydantoin-based antivirulence agents as promising leads for non-bactericidal anti-infective strategies, potentially reducing selective pressure for antibiotic resistance.

**Figure 26:**
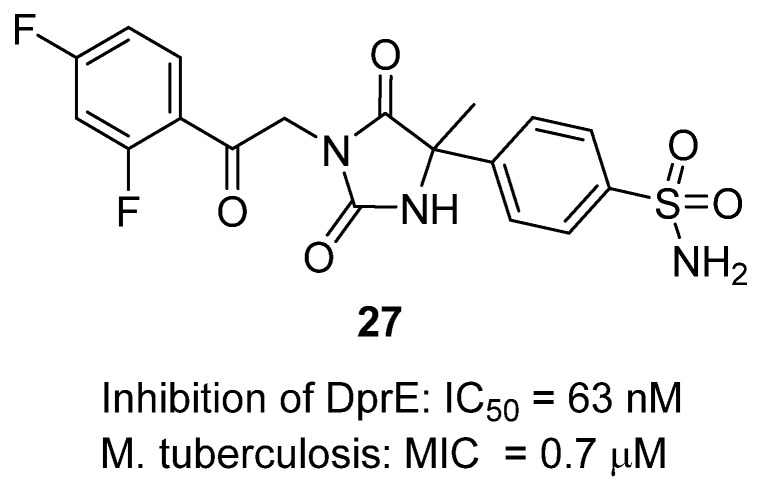
5-*p*-Sulfamoylphenylhydantoin **27**.

In a 2020 report, Balabon et al. described the optimization of hydantoins as potent inhibitors of decaprenylphosphoryl-β-d-ribose oxidase (DprE1), a validated target in *Mycobacterium tuberculosis* cell wall biosynthesis [46]. DprE1 catalyzes a key epimerization step in arabinan synthesis and is essential for bacterial survival, making it an attractive therapeutic target for drug-resistant tuberculosis. Within the optimized library, hydantoin **27** displayed excellent biochemical and cellular activity (Figure 26). It inhibited DprE1 with a pIC_50_ of 7.2 (~63 nM) and showed strong antimycobacterial potency with a MIC of 0.7 µM against *M. tuberculosis* H37Rv, comparing favorably with isoniazid (MIC = 1.8 µM). Structurally, **27** combines a hydantoin core with a 2,4-difluorophenyl ketone side chain and a para-sulfonamide-substituted aryl moiety, a motif that optimization identified as critical for balancing solubility, potency, and metabolic stability. SAR exploration revealed that electron-withdrawing substituents on ring B, particularly fluorine patterns such as 3,4-difluoro, enhanced both DprE1 affinity and whole-cell activity, while bulkier or electron-donating groups reduced potency. Importantly, **27** and close analogues showed no significant cytotoxicity in HepG2 cells (IC_50_ > 100 µM), no hERG channel inhibition, and good microsomal stability, suggesting a favorable safety profile. Proof-of-concept in vivo efficacy was demonstrated with related analogues in an acute murine tuberculosis infection model, where oral dosing reduced lung bacterial loads. Overall, **27** exemplifies the hydantoin scaffold’s promise as a noncovalent, selective, and drug-like class of DprE1 inhibitors, offering a strong lead for further preclinical development of next-generation antitubercular agents.

**Figure 27:**
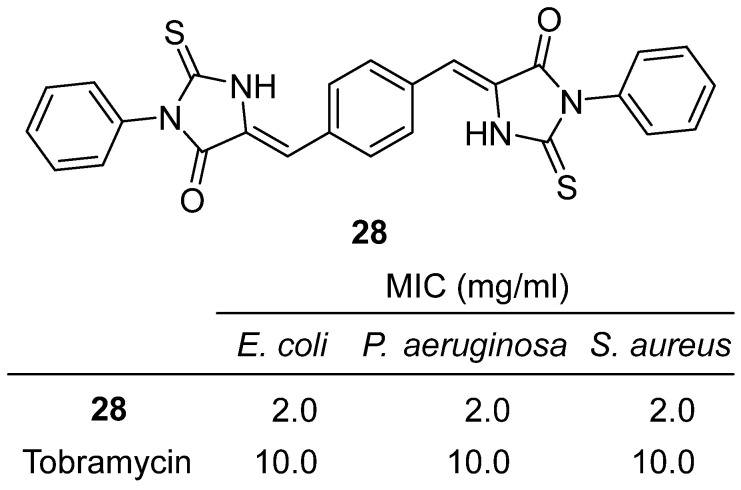
Symmetrical bis-thiohydantoin **28**.

In 2024, Khodair and colleagues synthesized and characterized a series of new bis-hydantoin and bis-thiohydantoin derivatives, evaluating their antibacterial activity and molecular interactions [47]. **28**, a bis-thiohydantoin featuring two 3-phenyl-2-thioxoimidazolidin-4-one units linked via a 1,4-phenylene bridge, was obtained as orange crystals and confirmed by NMR, IR, and elemental analysis (Figure 27). **28** exhibited moderate antibacterial effects, with inhibition zone diameters of 3.0 mm against *E. coli* and *S. aureus*, and 2.0–3.0 mm against *P. aeruginosa* and *E. faecalis*. Its minimum inhibitory concentration (MIC) was 2.0 mg/mL for *E. coli* and *S. aureus*, and minimum bactericidal concentration (MBC) was 2.4 mg/mL and 2.1 mg/mL, respectively—showing greater potency than the reference antibiotic tobramycin (MIC = 10 mg/mL). Molecular docking studies revealed that **28** binds strongly to the active site of bacterial DNA gyrase (PDB: 6RKU), with a binding affinity of −9.6 kcal/mol. While **28** did not form direct hydrogen bonds, its high affinity was attributed to strong interactions with key residues (SER-111, SER-116, ALA-117, MET-120, TYR-86, ASP-87, ARG-121, among others), supporting its role as a DNA gyrase inhibitor. The presence of phenyl substituents at the thiohydantoin core and the rigid bis-phenylene linkage contribute to the compound’s planarity and extended conjugation, which are favorable for protein binding and antibacterial activity. Compared to other analogues, **28**’s moderate activity suggests that further optimization of substituents and electronic properties could enhance potency.

**Figure 28:**
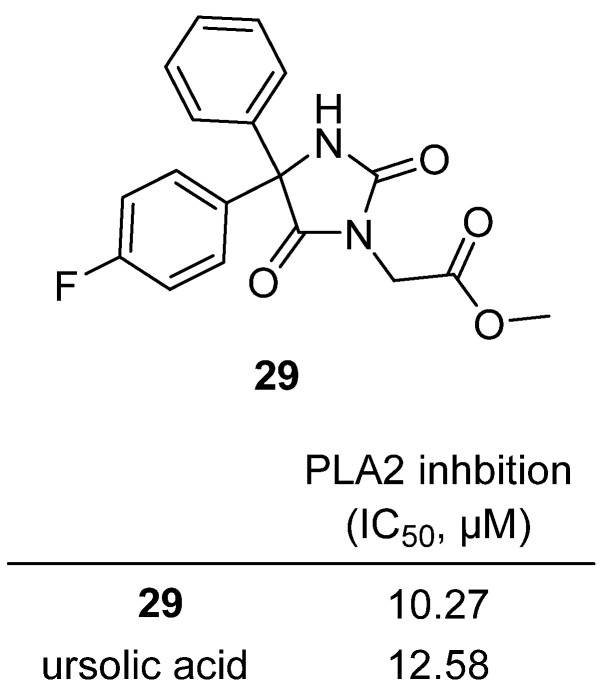
Diphenylhydantoin **29**.

In a 2023 study published in *Chemical Physics Impact*, Theodore and colleagues synthesized and characterized a series of N-substituted hydantoin derivatives, evaluating their antimicrobial and phospholipase A2 (PLA2) inhibitory activities [48]. Among these, diphenylhydantoin **29**—a methyl 2-(4-(4-fluorophenyl)-2,5-dioxo-4-phenylimidazolidin-1-yl)acetate—emerged as a standout lead [48] (Figure 28). **29** exhibited potent broad-spectrum antibacterial and antifungal activity, with MIC values of 11.7–13.5 µM against Gram-positive (*S. aureus*, *B. subtilis*), Gram-negative (*E. coli*), and fungal (*C. albicans*) strains, matching or surpassing reference drugs fluconazole and streptomycin. In addition, **29** was a strong inhibitor of the PLA2 enzyme (IC_50_ = 10.27 µM), outperforming the reference inhibitor ursolic acid (IC_50_ = 12.58 µM). Structure–activity relationship (SAR) analysis indicated that the presence of a para-fluoro group on one phenyl ring and a methyl acetate substituent at N-1 of the hydantoin core significantly enhances both antimicrobial and PLA2 inhibitory potency. Drug-likeness predictions confirmed that **29** meets Lipinski’s rule of five, suggesting good oral bioavailability and favorable physicochemical properties.

**Figure 29:**
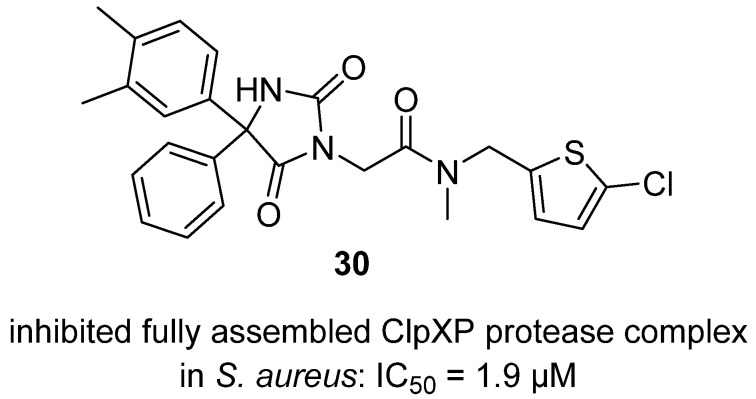
Diphenylhydantoinyl acetamide **30**.

In a 2019 study, Fetzer and colleagues reported the discovery and characterization of compound **1**, a stereospecific hydantoin analog that potently inhibits the fully assembled ClpXP protease complex in *Staphylococcus aureus* (IC_50_ = 1.9 µM), without affecting the individual ClpP peptidase or ClpX chaperone activities [49]. Hydantoin **30** acts through reversible binding to ClpP, stabilizing its structure without covalent modification or disruption of oligomeric assembly, and SAR analysis revealed that sterically demanding groups at the hydantoin core are essential for activity, with only one enantiomer being active, thus establishing **30** as a unique tool for modulating bacterial proteostasis and a promising lead for further therapeutic development targeting ClpXP-dependent pathways (Figure 29).

### 2.4. Antiviral

**Figure 30:**
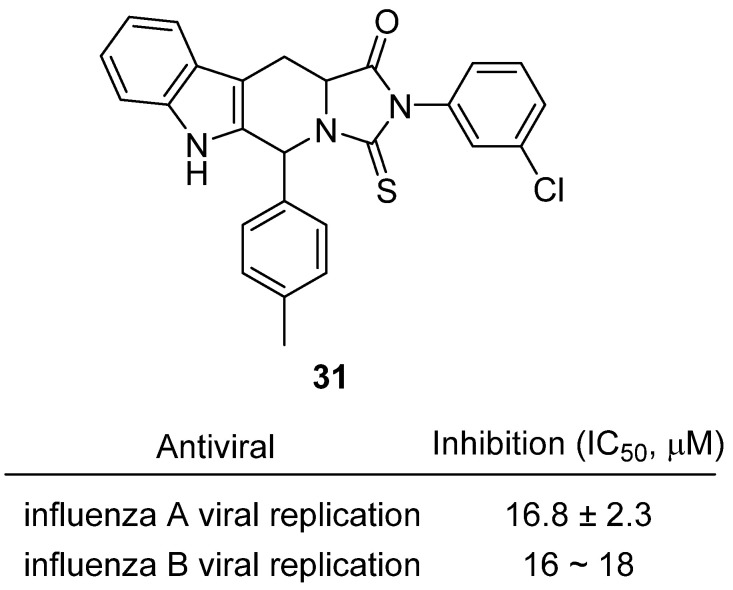
Tetrahydro-β-carboline-fused hydantoin **31**.

In 2025, Khachatryan et al. reported the synthesis of tetrahydro-β-carboline–thiohydantoin hybrids as a novel class of antivirals targeting influenza virus [50]. These compounds were designed by fusing the indole-containing tetrahydro-β-carboline scaffold, known for diverse bioactivities, with the thiohydantoin ring, to yield rigid heterocyclic systems capable of engaging viral or host factors. From a library of 23 analogues, nearly half displayed antiviral selectivity indices (SI ≥ 10), substantially exceeding the reference drug rimantadine, and several showed dual activity against both influenza A (H1N1, A/Puerto Rico/8/34) and influenza B (B/Malaysia/2506/04) strains. A representative compound, hydantoin **31**, inhibited influenza A viral replication with an IC_50_ of 16.8 ± 2.3 µM and demonstrated similar potency against influenza B (IC_50_ ≈ 16–18 µM), while maintaining low cytotoxicity (CC_50_ > 650 µM, SI~39) (Figure 30). Time-of-addition assays indicated that these hybrids act at the late stage of the viral cycle (4–6 h post-infection), likely interfering with virion assembly or budding, but without direct neuraminidase inhibition, as confirmed by enzymatic assays. This mechanism differentiates them from neuraminidase inhibitors (zanamivir, oseltamivir) and M2 blockers (amantadine, rimantadine), suggesting an alternative mode of action less vulnerable to existing resistance.

**Figure 31:**
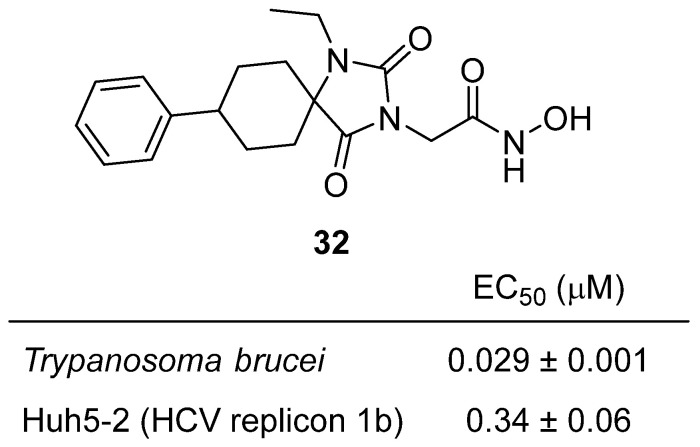
Hydroxamate-tethered hydantoin **32**.

In 2023, Pardali and colleagues reported the discovery of lipophilic hydroxamates based on spirocarbocyclic hydantoin scaffolds as dual-acting agents with both antiviral and trypanocidal activity [51]. The design strategy combined the hydantoin core, known for its metabolic stability and hydrogen-bonding capacity, with hydroxamate moieties to chelate metal ions, while incorporating bulky spirocarbocyclic substituents to enhance lipophilicity and membrane penetration. Among the synthesized library hydantoin **32** emerged as the standout analogue (Figure 31). It displayed exceptional potency against *Trypanosoma brucei* with an EC_50_ of 29 nM, marking it as a strong lead for anti–African trypanosomiasis therapy, while also showing low-nanomolar to submicromolar antiviral activity. In HCV replicon assays (Huh5-2, replicon 1b), **32** inhibited viral replication with an EC_50_ of 0.34 ± 0.05 µM, demonstrating dual antiparasitic and antiviral efficacy. SAR studies revealed that the spirohydantoin framework provided conformational rigidity, improving target binding and selectivity, while the hydroxamate substituent was critical for bioactivity. These results highlight **32** as a promising dual-action hydantoin-based lead, offering both potent trypanocidal and antiviral effects, and exemplifying the potential of spirocarbocyclic hydantoins as multifunctional therapeutic scaffolds.

### 2.5. Neurologic

**Figure 32:**
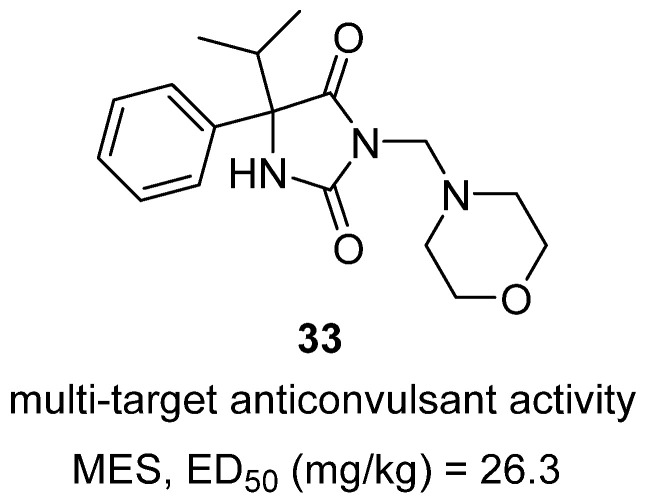
3-Morpholinomethyl hydantoin **33**.

In a 2025 *ChemMedChem* article, Czopek and colleagues reported the synthesis and evaluation of novel hybrid imidazolidine-2,4-dione (hydantoin) derivatives containing a morpholine moiety as potential multi-target anticonvulsants [52]. The design rationale was to merge pharmacophoric features of phenytoin (a classic sodium channel blocker) and imepitoin (a morpholine-containing AED) into single molecules with improved therapeutic breadth. Among the series, compound **33**, a 5-isopropyl-3-(morpholinomethyl)-5-phenylhydantoin, emerged as one of the most promising analogues (Figure 32). In the maximal electroshock seizure (MES) model, **33** protected animals with an ED_50_ of 26.3 mg/kg, while also demonstrating robust efficacy in the 6 Hz test, a pharmacoresistant focal seizure model, with ED_50_ values of 11.1 mg/kg (32 mA) and 40.9 mg/kg (44 mA). This spectrum was broader than phenytoin, which is inactive in the 6 Hz model, and nearly 1.5-fold more effective than levetiracetam in the 6 Hz (32 mA) test. Importantly, safety evaluations showed no hepatotoxicity in HepG2 cells, and no acute motor impairment at doses ≤100 mg/kg in rotarod assays, though higher doses caused some neurotoxicity. Overall, **33** demonstrates potent, broad-spectrum anticonvulsant activity with a favorable safety profile, establishing hydantoin–morpholine hybrids as promising scaffolds for further preclinical development in epilepsy.

**Figure 33:**
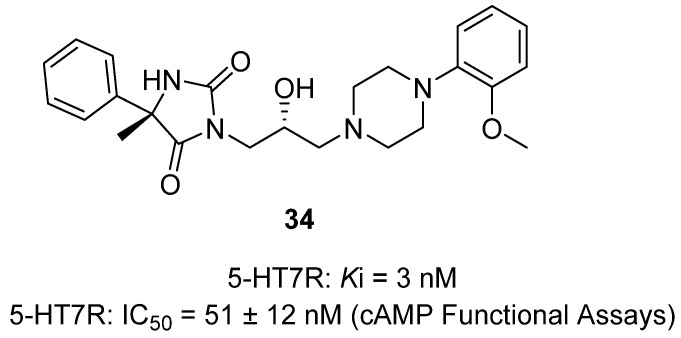
Chiral Phenylpiperazine-hydantoin conjugate **34**.

In a 2024 *ACS Chemical Neuroscience* study, Kucwaj-Brysz and colleagues presented the synthesis and pharmacological evaluation of stereochemically defined hydantoin–piperazine derivatives as potent 5-HT7 receptor (5-HT7R) antagonists [53]. The 5-HT7R has emerged as a promising target in CNS disorders including depression, anxiety, schizophrenia, and neurodegeneration, yet no selective drugs have reached clinical approval. The authors previously identified racemic hydantoin–phenylpiperazines with sub-100 nM affinity, but here they resolved and characterized the individual stereoisomers for the first time. X-ray crystallography confirmed absolute configurations at the hydantoin C5 and linker C7 stereogenic centers, and optically pure isomers were synthesized via resolution and stereoselective epoxide ring opening. Biological profiling revealed strong stereochemical dependence: hydantoin **34** (5S,7R configuration as shown in Figure 33. displayed high binding affinity (Ki = 3 nM) and potent functional antagonism in cAMP assays (IC_50_ = 51 ± 12 nM), with marked selectivity over D2, 5-HT1A, and 5-HT2A receptors. In vivo, the racemic parent and its (5S,7R) stereoisomer **34** significantly reduced immobility in the forced swim test (antidepressant-like effect) and increased punished crossings in the four-plate test (anxiolytic-like effect) in mice, demonstrating CNS efficacy at low doses. Molecular modeling and MD simulations indicated that avoiding persistent contact with residue I3 × 29 in the 5-HT7R binding pocket was a key determinant of high affinity, while favorable salt-bridge interactions with D3 × 32 stabilized the ligand.

**Figure 34:**
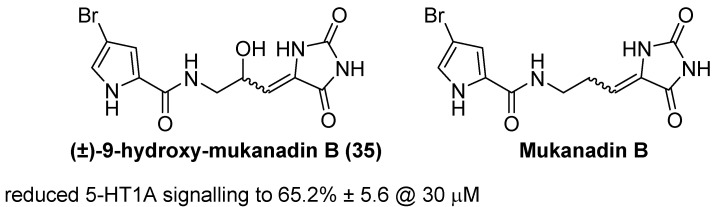
(±)-9-hydroxy-mukanadin B (**35**).

In a 2024 *ChemMedChem* report, Rees and colleagues explored mukanadin B, D, and F analogues as potential antagonists of serotonergic signaling, focusing on their interactions with the 5-HT1A receptor [54]. The mukanadin family are marine bromopyrrole alkaloids with a hydantoin moiety, and earlier studies suggested that certain members could interfere with serotonin pathways. In this work, thirteen new derivatives and several natural products were synthesized with modifications to the pyrrole ring, central linker, and hydantoin core. Among the tested molecules, (±)-9-hydroxy-mukanadin B (**35**) demonstrated moderate antagonism: in IP1 accumulation assays using 5-HT1A-transfected Cos-7 cells, it reduced 5-HT1A signaling to ~65% of control (65.2% ± 5.6) at 30 µM (Figure 34). While weaker than the parent mukanadin B (69.3%) and less potent than some newly synthesized analogues, **35** nonetheless validated the role of the hydantoin–pyrrole pharmacophore in serotonin antagonism. SAR analysis indicated that pyrrole substitution patterns were well tolerated, but saturation of the central linker and replacement of the hydantoin with a triazole ablated activity, underscoring the importance of the unsaturated linker and hydantoin ring for 5-HT1A modulation. Overall, **35** represents a proof-of-concept natural product analogue, supporting the idea that marine-derived hydantoin scaffolds can yield non-cytotoxic serotonergic antagonists with potential application in mood disorder therapy.

### 2.6. Antiprotozoan

**Figure 35:**
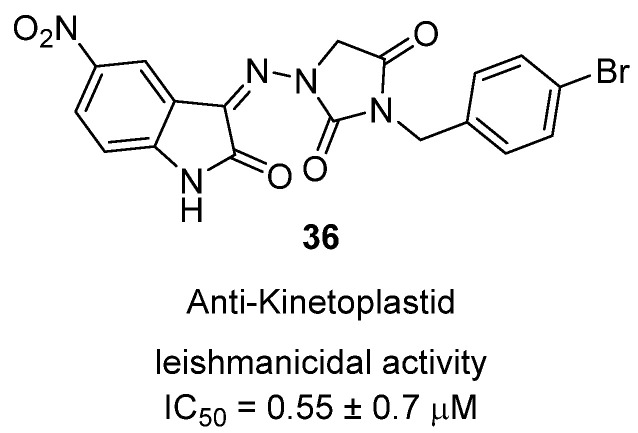
5-Nitroisatinylated hydantoin **36**.

In 2025, Sechoaro et al. reported the synthesis and biological evaluation of isatinylhydantoin derivatives as novel anti-kinetoplastid agents, targeting both trypanosomes and Leishmania species [55]. The series was prepared in two steps: condensation of 1-aminohydantoin with various isatins to form isatinylhydantoin intermediates, followed by benzylation at N-3. Among these, hydantoin **36**, bearing a 5-nitroisatin core and a p-bromobenzyl substituent, displayed particularly strong activity against kinetoplastid parasites (Figure 35). It showed potent leishmanicidal activity with an IC_50_ of 0.55 ± 0.7 µM against *Leishmania donovani* amastigotes, surpassing amphotericin B in selectivity indices, and was also active against multiple *Trypanosoma* species. Importantly, cytotoxicity testing against mammalian Vero and THP-1 cells revealed low host toxicity (CC_50_ > 100 µM), translating to high selectivity (SI > 180). Mechanistically, the activity is thought to stem from inhibition of parasite cysteine proteases such as cruzipains and cathepsin L-like enzymes, which are critical for parasite survival and virulence. Notably, while some isatinylhydantoins showed strong in vitro trypanocidal activity, they lacked in vivo efficacy in mouse models, highlighting the importance of pharmacokinetic properties. In contrast, **36** stands out as an early antileishmanial lead, combining potency, selectivity, and low cytotoxicity, and represents a promising candidate for further development in the treatment of neglected tropical diseases such as visceral leishmaniasis and African trypanosomiasis.

**Figure 36:**
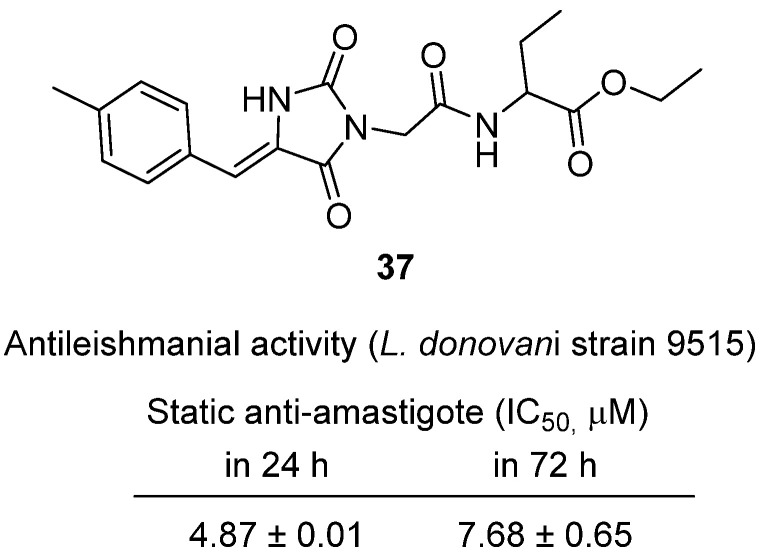
5-Arylidenehydantoinyl acetamide **37**.

In 2025, Aucamp et al. investigated arylidene analogues of glitazone, rhodanine, and hydantoin scaffolds for their potential as antiprotozoal agents, with a focus on antileishmanial efficacy [56]. Within this series, hydantoin **37**, an arylidene-glitazone derivative, was identified as one of the most promising leads (Figure 36). Against antimony-resistant *Leishmania donovani* strain 9515, **37** demonstrated potent anti-amastigote activity with IC_50_ values of 4.87 ± 0.01 µM (static, 24 h) and 7.68 ± 0.65 µM (72 h). These data suggest sustained activity with only minor parasite recovery, indicative of a static-to-cidal effect profile. Importantly, cytotoxicity testing in mammalian Vero and THP-1 cells yielded CC_50_ > 100 µM, resulting in a high selectivity index (SI > 100), surpassing the threshold proposed for early antileishmanial leads. Although most analogues in the study lacked significant activity against *Trypanosoma* species or showed no antidiabetic cross-activity, **37** emerged as a selective antileishmanial candidate, highlighting the pharmacological value of glitazone-based arylidene hybrids.

**Figure 37:**
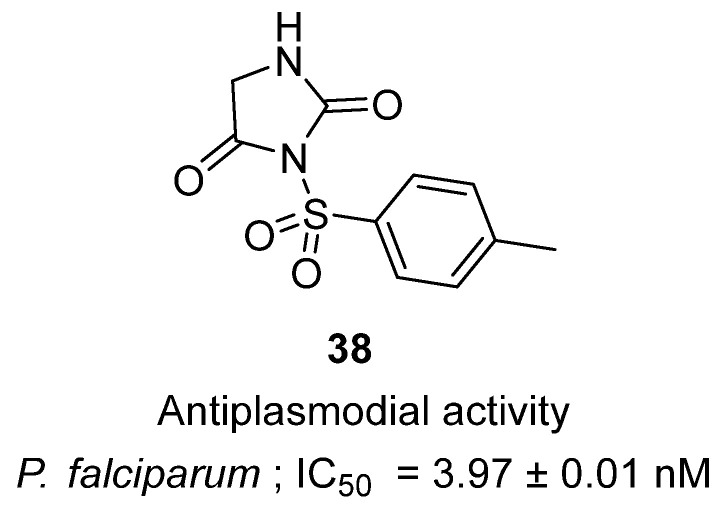
3-*N*-Toluenesulfonylhydantoin (**38**).

In 2024, Chin and co-workers described the synthesis of new 3-substituted and 3,5-disubstituted hydantoins as potential antimalarial agents targeting *Plasmodium falciparum* [57]. Within this series, hydantoin **38**, featuring a p-toluenesulfonyl substituent at N-3, stood out as the most active (Figure 37). Biologically, **38** demonstrated potent inhibition of *P. falciparum* 3D7 strain with an IC_50_ of 3.97 ± 0.01 nM, which was approximately three-fold more potent than chloroquine (IC_50_ = 11.72 nM). Mechanistically, the introduction of the sulfonamide group was critical, as it enhanced hydrogen-bonding capacity and polarity while maintaining lipophilicity, supporting high affinity for the parasite target. Other hydantoin derivatives in the article showed only moderate to weak activity (27 µM to >700 µM), underscoring the unique contribution of the sulfonyl group in **38**. Importantly, **38** displayed no detectable cytotoxicity toward normal human lung fibroblast MRC-5 cells (IC_50_ > 100 µM), yielding an excellent selectivity index (>25,000). These findings establish **38** as a highly promising antiplasmodial lead, combining nanomolar potency, selectivity, and a safe cytotoxicity profile, and highlighting the hydantoin scaffold—particularly sulfonamide-modified analogues—as a strong platform for future malaria drug development.

**Figure 38:**
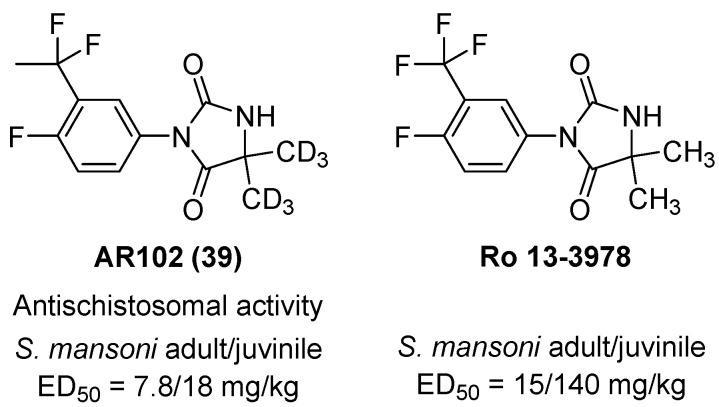
5,5-bis(methyl-*d*_3_)hydantoin **39**.

In 2024, Vennerstrom and colleagues reported the optimization of aryl hydantoins as next-generation antischistosomal agents, culminating in the discovery of the single-dose candidate AR102 (**39**) [58] (Figure 38). Early hydantoin leads such as Ro 13-3978 demonstrated potent in vivo activity against *Schistosoma mansoni* but were limited by antiandrogenic side effects (due to similarity to nilutamide) and rapid metabolic clearance. Two critical structural modifications overcame these liabilities: (i) replacing the aryl trifluoromethyl substituent with a difluoroethyl group abolished antiandrogenic activity, and (ii) deuteration of the gem-dimethyl group enhanced metabolic stability by reducing phase I clearance. The resulting analogue AR102 exhibited ED_50_ values of 7.8 mg/kg (adult *S. mansoni*) and 18 mg/kg (juvenile *S. mansoni*), with similarly potent efficacy against *S. haematobium* (ED_50_ = 8.5 mg/kg) and *S. japonicum* (ED_50_ = 6.3 mg/kg). These values far surpass praziquantel, which shows ED_50_ > 170 mg/kg and poor juvenile-stage activity. Pharmacokinetically, **39** displayed high oral bioavailability (~80%), long half-lives across species, and low clearance, enabling single-dose curative potential. Importantly, it showed no AR antagonism up to 100 µM, no hERG inhibition, no CYP450 liabilities, and no mutagenicity in Ames or mouse lymphoma assays. Toxicology studies identified a rat NOAEL of 25 mg/kg and a dog NOAEL of >150 mg/kg for single oral dosing, with reversible histopathological findings at higher doses.

**Figure 39:**
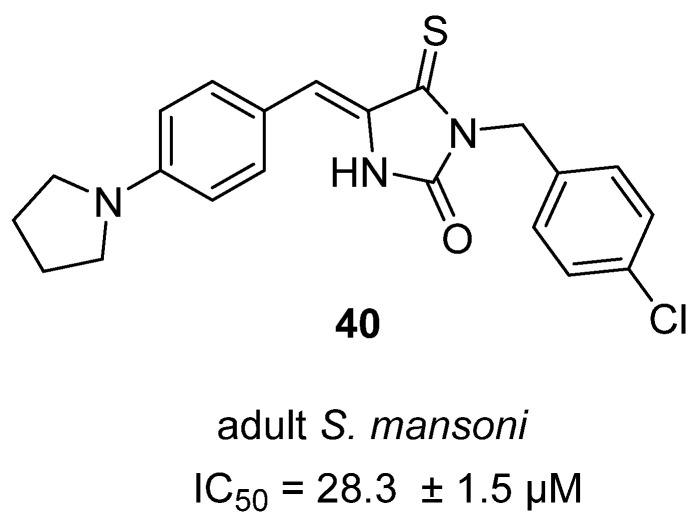
1-(4-chlorobenzyl)-4-(4-(pyrrolidin-1-yl)benzylidene)thiohydantoine **40**.

In a 2024 study, Almeida Júnior and colleagues evaluated the antiparasitic potential of hydantoin and thiohydantoin compounds against *Schistosoma mansoni*, with a particular focus on a thiohydantoin **40** [59] (Figure 39). **40** demonstrated the highest schistosomicidal activity among the tested compounds, causing 100% mortality of adult *S. mansoni* worms within 24 h at a concentration of 50 µM (IC_50_ = 28 µM), outperforming other analogues and showing rapid, dose-dependent effects on parasite motility and survival. Ultrastructural analysis by scanning electron microscopy revealed that **40** induced significant damage to the worm’s tegument, including blister formation, erosion, and destruction of tubercles and spicules, suggesting a direct effect on the parasite’s protective surface. In addition, **40** exhibited moderate cytotoxicity in mammalian cell lines (CC_50_ values: 49.5–74.6 µM across J774 macrophages, V79 fibroblasts, Vero, and HepG2 cells) and was non-hemolytic at the highest tested concentration. Mechanistic studies showed that **40** interacts moderately with DNA via a static quenching mechanism, which may contribute to its antiparasitic action.

### 2.7. Metabolic

**Figure 40:**
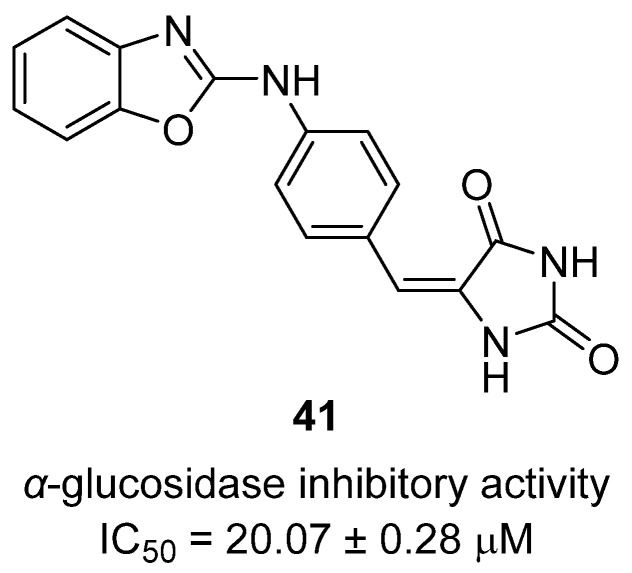
4-(Benzoxazolylamino)phenylenehydantoin **41**.

In 2021, Singh and co-workers reported the design and synthesis of benzoxazolyl-linked benzylidene derivatives incorporating thiazolidinedione, rhodanine, hydantoin, and thiohydantoin scaffolds as potential antidiabetic agents targeting α-glucosidase [60]. Biological screening against α-glucosidase (using acarbose as a reference, IC_50_ = 15.01 µM) revealed that hydantoin-containing analogues also showed activity, though weaker. *para*-Substituted hydantoin **41** and *meta*-substituted hydantoin exhibited IC_50_ values of 20.07 ± 0.28 µM and 20.93 ± 0.66 µM, respectively, indicating moderate inhibitory potential compared to acarbose (Figure 40). Docking studies using the α-glucosidase crystal structure (PDB 3TOP) supported the bioassay data, showing that the hydantoin NH and carbonyl groups formed hydrogen bonds with Asp1526, Arg1510, and Trp1523 residues, but with fewer interactions and weaker Gold scores compared to rhodanine analogues. Importantly, SAR analysis showed that meta-substitution generally improved binding affinity relative to para-substitution, though hydantoins remained less potent than rhodanine congeners.

**Figure 41:**
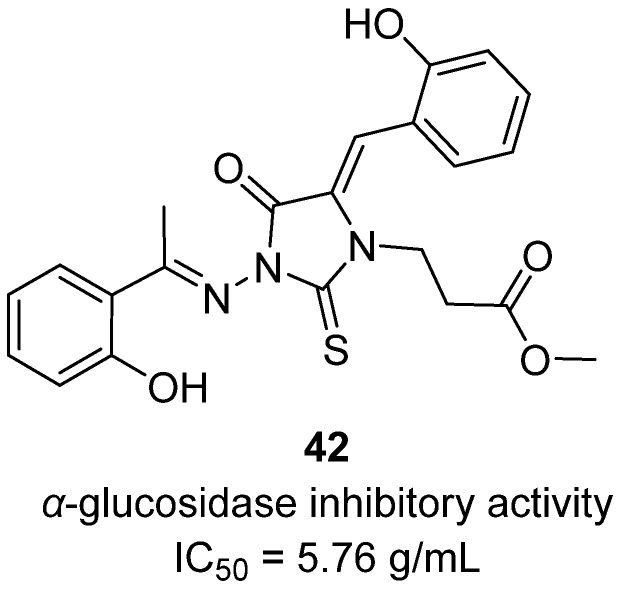
1,3,5-Trisubstituted hydantoin **42**.

In a 2022 article and colleagues designed and synthesized a new class of 1,3,5-trisubstituted-2-thioxoimidazolidin-4-one derivatives (trisubstituted thiohydantoins) to evaluate their antidiabetic potential through α-glucosidase and α-amylase inhibition, antioxidant activity, and cytotoxicity [61]. Within this series, hydantoin **42** emerged as one of the most promising candidates (Figure 41). Structurally, it incorporates a vinylphenol moiety at the C5 position, a 2-hydroxyphenylethylidene substituent at N3, and a methylpropionate side chain at N1, allowing multiple hydrogen-bond donors and acceptors to engage the catalytic residues of the target enzymes. Biologically, **42** inhibited α-glucosidase with an IC_50_ of 5.76 µg/mL, comparable to the standard drug acarbose (IC_50_ = 5.76 µg/mL), and also showed potent inhibition of α-amylase with an IC_50_ of 0.21 µg/mL, nearly twice as strong as acarbose (IC_50_ = 0.39 µg/mL). Beyond enzyme inhibition, **42** demonstrated antioxidant capacity by scavenging DPPH free radicals (IC_50_ = 51.75 µg/mL, close to Trolox at 58.09 µg/mL) and produced low levels of ROS (132.4 pg/mL) compared to the ROS-inducing drug celecoxib (171.6 pg/mL), indicating redox safety. Cytotoxicity evaluation against human lung fibroblasts (WI-38) showed low toxicity (IC_50_ = 88.54 µg/mL), consistent with a favorable therapeutic window.

**Figure 42:**
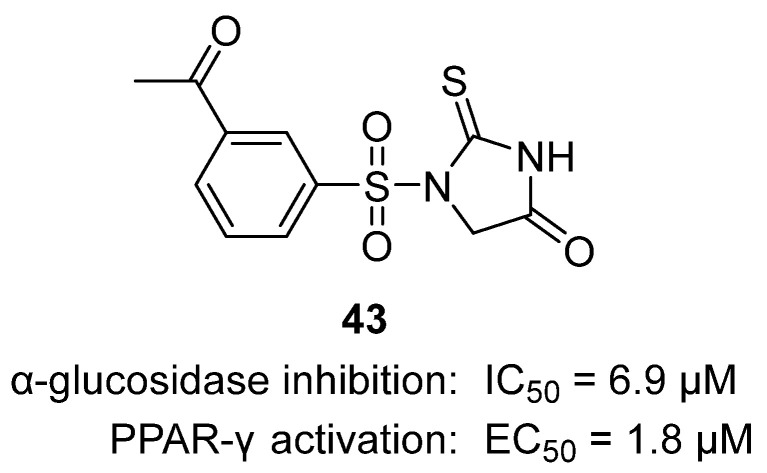
1-N-Phenylsulfonyl thiohydantoin **43**.

In 2025, Bukhari and coworkers synthesized and evaluated a series of thiohydantoin derivatives for antidiabetic effects [62]. Among them, thiohydantoin **43** exhibited the most favorable profiles (Figure 42). Structurally, **43** features a thiohydantoin core with meta-acetyl substituent, enhancing hydrogen bonding and π–π interactions with catalytic residues of α-glucosidase and α-amylase, as confirmed by molecular docking (−6.9 kcal/mol and −7.8 kcal/mol, respectively). **43** demonstrated meaningful in vitro inhibition against α-glucosidase (IC_50_ = 129.40 µg/mL) and α-amylase (IC_50_ = 128.90 µg/mL), moderate antioxidant activity (DPPH IC_50_ = 39.7 µg/mL), and moderate cytotoxicity (3T3 fibroblast IC_50_ = 598.3 µg/mL). In vivo, **43** lowered fasting blood glucose in diabetic rats by 28.9%, reduced HbA1C and suppressed diabetes-related weight loss, while improving lipid profiles by lowering LDL and triglycerides and elevating HDL. Thiohydantoin **43** stands out as a multifunctional antidiabetic and antihyperlipidemic lead compound, warranting continued pharmacokinetic and mechanistic investigation for clinical translation.

**Figure 43:**
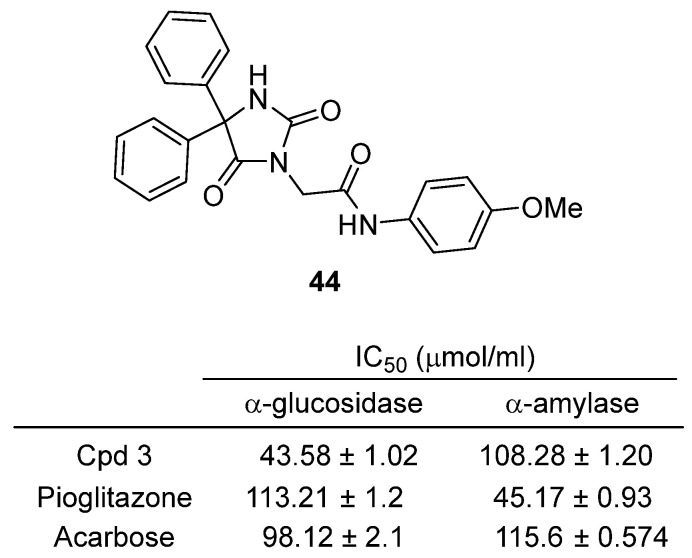
(5,5-diphenyl)hydantoinylacetamie **44**.

In 2025, Guerrab and colleagues reported the design, synthesis, and comprehensive profiling of a phenytoin-derived hydantoin compound **44** (2-(2,5-dioxo-4,4-diphenylimidazolidin-1-yl)-N-(4-methoxyphenyl)acetamide), as a promising antihyperglycemic agent [63] (Figure 43). **44** was synthesized via N-alkylation of phenytoin with an arylacetamide, and its structure was confirmed by NMR, IR, mass spectrometry, and single-crystal X-ray diffraction, revealing a W-shaped conformation stabilized by multiple hydrogen bonds and aromatic interactions. In vitro, **44** inhibited α-glucosidase (IC_50_ = 43.58 ± 1.02 µM) and α-amylase (IC_50_ = 108.28 ± 1.20 µM), outperforming or matching the standard drug acarbose. In an oral glucose tolerance test (OGTT) in mice, **44** (50 mg/kg) significantly reduced postprandial blood glucose, showing greater efficacy than acarbose. Acute toxicity studies revealed no adverse effects at 2 g/kg, indicating a favorable safety profile. Antioxidant assays (DPPH, ABTS, FRAP) showed that **44** possesses moderate antioxidant activity, though less potent than ascorbic acid.

### 2.8. Antiinflammatory and Immunomodulatory

**Figure 44:**
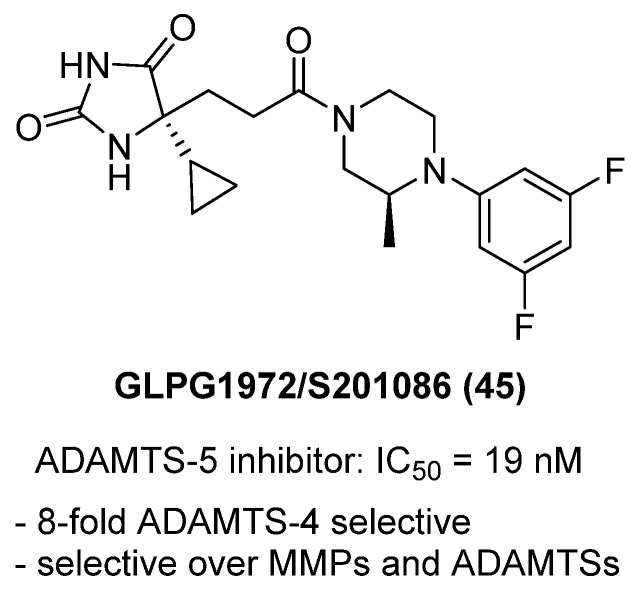
p-Fluorophenylpiperazine-tethered hydantoin **45**.

Brebion and colleagues described the discovery of GLPG1972/S201086 (**45**), a potent, selective, and orally bioavailable ADAMTS-5 inhibitor developed as a potential disease-modifying osteoarthritis drug (DMOAD) in 2021 [64] (Figure 44). ADAMTS-5 is the major aggrecanase driving cartilage degradation in osteoarthritis, and its inhibition has been validated genetically and pharmacologically as a disease-modifying strategy. **45** originated from a hydantoin hit identified in an HTS campaign, which was optimized through iterative SAR studies to enhance potency, metabolic stability, and selectivity. Structurally, **45** features a hydantoin zinc-binding group (ZBG) with a cyclopropyl substituent at C-5 and a para-fluorophenyl–piperazine amide moiety occupying the S′1 pocket of ADAMTS-5. This design enabled strong zinc coordination, hydrogen bonding with Glu411 and Gly380, and hydrophobic interactions with Leu443 and Met381, as confirmed in a cocrystal structure (PDB 6YJM). Biochemically, **45** inhibited human ADAMTS-5 with an IC_50_ of 19 nM, while maintaining 8-fold selectivity over ADAMTS-4 and >100-fold selectivity over a wide panel of MMPs and ADAM proteases. In mouse cartilage explant assays, it reduced glycosaminoglycan release with IC_50_ < 1.5 µM, confirming functional inhibition in a disease-relevant model. Pharmacokinetic studies showed high oral bioavailability (97% in dogs, 58% in rats), moderate plasma protein binding, good hepatocyte stability, and no CYP450 or hERG liabilities. Toxicological studies confirmed no mutagenicity and low risk for QT prolongation. **45** progressed into Phase 2 clinical trials (NCT03595618) in knee osteoarthritis patients, representing one of the most advanced ADAMTS-5 small-molecule inhibitors reported.

**Figure 45:**
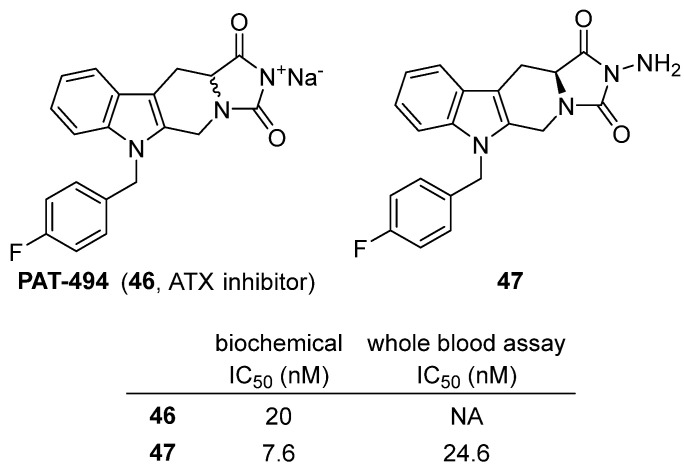
Polycyclic hydantoin **46** and **47**.

In 2022, Roy and colleagues reported the structure-based discovery of PAT-494 (**46**) and optimized analogues as potent autotaxin (ATX) inhibitors [65]. ATX is a secreted lysophospholipase D (lysoPLD) that converts lysophosphatidylcholine (LPC) into lysophosphatidic acid (LPA), a bioactive lipid involved in fibrosis, inflammation, neuropathic pain, rheumatoid arthritis, cardiovascular diseases, and cancer. The ATX–LPA signaling axis is thus an attractive therapeutic target. PAT-494 (**46**), a tetracyclic hydantoin-containing compound, was initially identified as a mixed-mode ATX inhibitor (Figure 45). Importantly, the S-enantiomer was twice as active as the R-enantiomer, highlighting stereochemical dependence. Biologically, **46** showed IC_50_ of 20 nM in enzymatic assays, while the optimized **47** (an aminohydantoin analogue derived from L-tryptophan) demonstrated superior potency (IC_50_ = 7.6 nM biochemically, 24.6 nM in whole blood assays), outperforming both **46** and the clinical reference PF-8380 (IC_50_ = 3.4 nM biochemical but 151 nM in whole blood). **47** was metabolically stable in human liver microsomes (CLint = 15.6 µL/min/mg, T_1_/_2_ = 113.2 min), moderately stable in rat liver microsomes, and orally bioavailable in rats (F = 14.3%). It was also non-cytotoxic (19% cell growth inhibition at 20 µM in HEK cells). Overall, PAT-494 validated the hydantoin chemotype as an effective ATX inhibitor, and further optimization yielded **47**, a safe, orally bioavailable, nanomolar inhibitor with strong whole-blood activity, demonstrating the translational potential of hydantoin-based ATX inhibitors for fibrotic, inflammatory, and oncological diseases.

**Figure 46:**
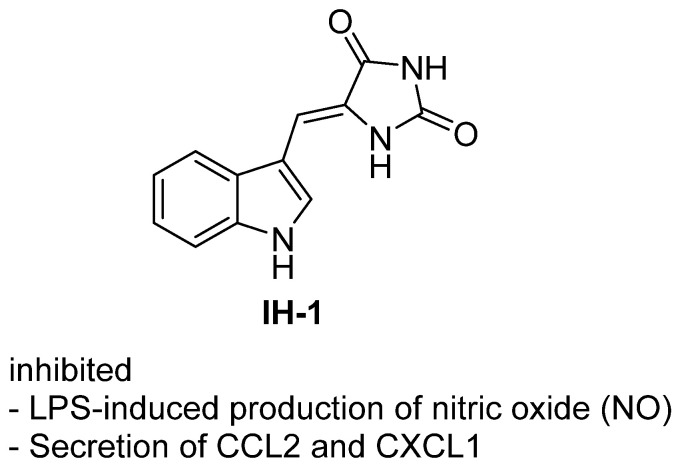
Indolidene hydantoin **48**.

In a 2021 study, Lin et al. reported the synthesis and biological evaluation of the indole-hydantoin derivative **48** (5-(1H-indole-3-ylmethylene)imidazolidine-2,4-dione) as a novel anti-inflammatory agent [66] (Figure 46). **48** was shown to significantly inhibit LPS-induced inflammatory responses in murine RAW264.7 macrophages by suppressing the production of nitric oxide (NO) and the secretion of chemokines CCL2 and CXCL1, through downregulation of iNOS, CCL2, and CXCL1 mRNA expression. Mechanistically, **48** specifically blocked the transactivation of NF-κB by inhibiting the phosphorylation of the p65 subunit at Ser276, without affecting IκBα degradation or NF-κB nuclear translocation. This led to reduced interaction between NF-κB p65 and the transcriptional coactivator CBP, thereby attenuating the expression of pro-inflammatory genes. **48** also reduced LPS-induced ROS production, but its anti-inflammatory effects were independent of antioxidant activity. Structure–activity relationship analysis revealed that the planar conformation of **48**, conferred by its conjugated double bond, was critical for activity, as related analogues lacking this feature were inactive.

### 2.9. Miscellaneous

**Figure 47:**
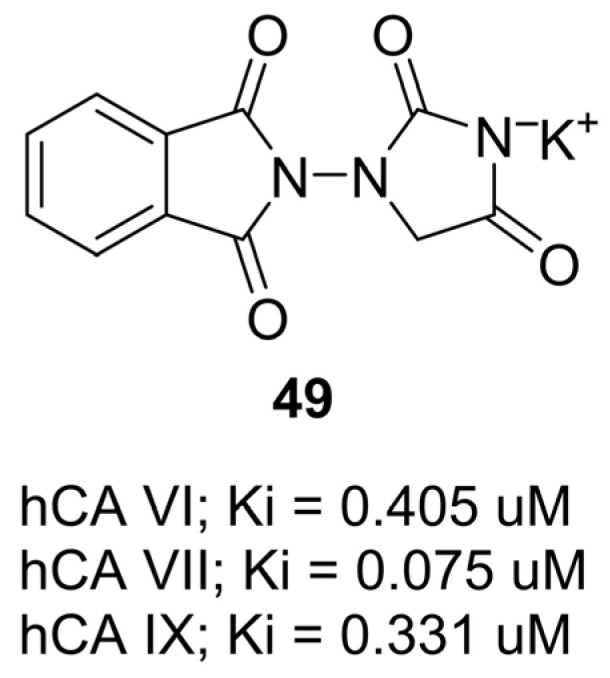
Phthalimide–hydantoin hybrid **49**.

In 2024, Abdoli and colleagues reported the design and biological evaluation of novel phthalimide–hydantoin hybrids as inhibitors of human carbonic anhydrase (hCA) isozymes [67]. Carbonic anhydrases play central roles in pH regulation, fluid secretion, and tumor progression, with isoforms hCA VI (secreted), hCA VII (neuronal), and hCA IX (tumor-associated) being especially relevant therapeutic targets. The compounds were synthesized by condensing activated phthalimides with 1-aminohydantoin, followed by benzylation to yield salts and neutral derivatives. Among the tested molecules, compound **49**, a potassium salt of a phthalimide–hydantoin hybrid, displayed the strongest inhibitory activity (Figure 47). It selectively inhibited hCA VII (Ki = 0.075 µM), hCA IX (Ki = 0.331 µM), and hCA VI (Ki = 0.405 µM), while showing no inhibition of the ubiquitous cytosolic isoform hCA I and only weak inhibition of hCA II. Docking studies indicated that the deprotonated imidic nitrogen of the hydantoin ring coordinated directly to the catalytic Zn^2+^ ion, completing its tetrahedral coordination sphere, while the phthalimide scaffold engaged in π–π stacking with the proton shuttle residue His64 and hydrogen bonding with Gln92 and Thr199. Importantly, **49** exhibited a favorable selectivity profile, being far more potent against hCA VII and IX than hCA II, suggesting potential for targeting neurological disorders and hypoxic tumors with reduced off-target effects.

**Figure 48:**
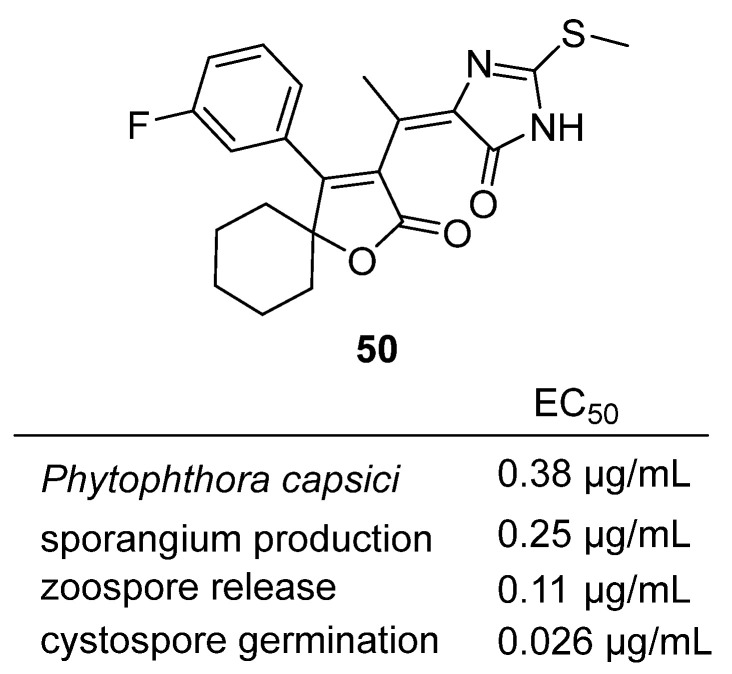
*S*-Methyl Thiohydantoin derivative **50**.

In 2023, Li et al. reported the design, synthesis, and biological evaluation of novel thiohydantoin analogues containing a spirocyclic butenolide scaffold for the control of plant-pathogenic oomycetes [68]. Among these, compound **50**, featuring a meta-fluorobenzene substituent, emerged as the most potent member of the series (Figure 48). In vitro, **50** exhibited outstanding inhibitory activity against *Phytophthora capsici*, with EC_50_ values of 0.38 μg/mL for mycelial growth, 0.25 μg/mL for sporangium production, 0.11 μg/mL for zoospore release, and 0.026 μg/mL for cystospore germination—significantly outperforming the commercial fungicide azoxystrobin. In vivo, **50** provided excellent protective and curative efficacy against *P. capsici* in pepper plants, achieving complete disease control at 100 μg/mL and maintaining >80% efficacy at 6.25 μg/mL, again surpassing azoxystrobin. Mechanistic studies revealed that **50** acts by binding to the Qo site of mitochondrial complex III, disrupting the respiratory chain and energy supply in the pathogen, as confirmed by enzyme inhibition and electron microscopy.

## 3. Perspectives

Hydantoins and thiohydantoins continue to stand out as privileged scaffolds in drug discovery, with applications extending far beyond their classical role as anticonvulsants. Recent studies demonstrate their broad utility across oncology, infectious diseases, cardiovascular and metabolic disorders, and central nervous system (CNS) therapy. This versatility arises from a favorable balance of hydrogen-bonding capacity, tunable lipophilicity, and stereochemical flexibility, enabling adaptation to diverse biological targets and disease-specific constraints.

From a disease-oriented perspective, several recurring insights emerge. In anticancer research, hydantoin derivatives have frequently been employed to engage kinases, apoptosis-related proteins, and other cancer-associated targets that benefit from precise spatial arrangement of functional groups. While high potency is often achieved, many studies reveal trade-offs between potency and selectivity, and metabolic liabilities linked to aromatic or highly lipophilic substitution patterns. These findings underscore the importance of context-dependent optimization rather than reliance on generalized SAR rules.

In antimicrobial applications, hydantoins are often valued for their synthetic accessibility and structural robustness, which facilitate rapid analogue generation and screening. Their success in this area appears to stem from the ability to balance polarity and lipophilicity within a compact scaffold. At the same time, challenges such as narrow activity spectra and susceptibility to resistance mechanisms are recurrent, highlighting the need for integration with modern antimicrobial design strategies.

CNS-oriented studies further illustrate the context-dependent strengths and limitations of hydantoins. The scaffold allows fine-tuning of physicochemical properties relevant to brain penetration and target engagement, yet CNS programs frequently expose narrow therapeutic windows. Optimization of solubility, metabolic stability, and off-target effects, therefore, remains critical for translational success.

Importantly, the medicinal chemistry value of hydantoins lies not in universally applicable SAR paradigms, but in their adaptability across therapeutic contexts. Compared with other commonly used privileged heterocycles, hydantoins offer a distinctive combination of conformational rigidity and chemical tunability, along with proven synthetic robustness. These features have enabled their incorporation into hybrid and multitarget designs and support their use in emerging drug discovery modalities.

The translational relevance of hydantoins is further supported by clinical-stage candidates such as GLPG1972 (ADAMTS-5 inhibitor), BAY-9835 (ADAMTS-7 inhibitor), and AR102 (antischistosomal agent), which collectively validate their drug-like potential across diverse disease areas. Looking ahead, the integration of structure-based design, AI-driven screening approaches, and polypharmacology-oriented strategies is expected to play a central role in unlocking new therapeutic opportunities for hydantoin-based scaffolds. Despite persistent challenges related to selectivity, metabolism, and disease-specific optimization, the chemical robustness and adaptability of hydantoins position them well to contribute to the next generation of small-molecule therapeutics targeting complex and treatment-resistant diseases.

## Data Availability

No new data were created or analyzed in this study. Data sharing is not applicable to this article.

## Abbreviations

The following abbreviations are used in this manuscript:

5-FU	5-Fluorouracil
5-HT	5-Hydroxytryptamine (Serotonin)
ABTS	2,2′-Azino-bis(3-ethylbenzothiazoline-6-sulfonic acid)
ADAMTS	A Disintegrin And Metalloproteinase with Thrombospondin Motifs
AED	Antiepileptic Drug
AR	Androgen Receptor
AT-1	Angiotensin II Type 1 Receptor
ATP	Adenosine Triphosphate
ATX	Autotaxin
BCL-2	B-Cell Lymphoma 2
Bcl-xL	B-Cell Lymphoma-Extra Large
CC50	50% Cytotoxic Concentration
CCL2	C-C Motif Chemokine Ligand 2 (MCP-1)
CDK	Cyclin-Dependent Kinase
ClpXP	ATP-Dependent Protease Complex ClpXP
CNS	Central Nervous System
CRPC	Castration-Resistant Prostate Cancer
CXCL1	C-X-C Motif Chemokine Ligand 1
DMOAD	Disease-modifying osteoarthritis drug
DPPH	2,2-Diphenyl-1-picrylhydrazyl (radical scavenging assay)
DprE1	Decaprenylphosphoryl-β-D-ribose oxidase
EBP	Emopamil binding protein
EGFR	Epidermal Growth Factor Receptor
ERK	Extracellular Signal-Regulated Kinase
FKBP5	FK506 Binding Protein 5
FRAP	Ferric Reducing Antioxidant Power (assay)
GI50	50% Growth Inhibition
GSK-3β	Glycogen Synthase Kinase-3 Beta
H1N1	Hemagglutinin 1 Neuraminidase 1 Influenza A Virus Subtype
hCA	human carbonic anhydrase
HCT-116	Human Colorectal Carcinoma Cell Line 116
HCV	Hepatitis C Virus
HDL	High-Density Lipoprotein
HePG-2	Human Hepatocellular Carcinoma Cell Line G2
HER2	Human Epidermal Growth Factor Receptor 2
hERG	human Ether-à-go-go-Related Gene
IDH1	Isocitrate Dehydrogenase 1
IR	Infrared
KLK3	Kallikrein Related Peptidase 3 (prostate-specific antigen, PSA)
LasR	*Pseudomonas aeruginosa* Quorum-Sensing Transcriptional Regulator
LDL	Low-Density Lipoprotein
LIHC	Liver Hepatocellular Carcinoma
LNCaP	Lymph Node Carcinoma of the Prostate
MBC	minimum bactericidal concentration
MCF-7	Michigan Cancer Foundation-7
MCL-1	Myeloid Cell Leukemia 1
MD	Molecular dynamics
MES	Maximal Electroshock Seizure (test)
MIC	Minimum inhibitory concentration
MRSA	Methicillin-Resistant *Staphylococcus aureus*
MS	Mass Spectrometry
NMR	Nuclear Magnetic Resonance (spectroscopy)
NO	nitric oxide
NOAEL	No Observed Adverse Effect Level
Nrf2	Nuclear Factor Erythroid 2-Related Factor 2
PDB	Protein Data Bank
Pgp	P-glycoprotein
PLA2	Phospholipase A2
PSA	Prostate-Specific Antigen
qRT-PCR	Quantitative Reverse Transcription Polymerase Chain Reaction
QT	QT Interval
RhlR	*Pseudomonas aeruginosa* Quorum-Sensing Transcriptional Regulator
ROS	Reactive Oxygen Species
SAR	Structure–Activity Relationship
TGI	Tumor Growth Inhibition
THP-1	Human Acute Monocytic Leukemia Cell Line
VCaP	Vertebral-Cancer of the Prostate (human prostate carcinoma cell line VCaP)
WT	Wild Type
